# CRL4-DCAF1 Ubiquitin Ligase Dependent Functions of HIV Viral Protein R and Viral Protein X

**DOI:** 10.3390/v16081313

**Published:** 2024-08-17

**Authors:** Ashley Dobransky, Mary Root, Nicholas Hafner, Matty Marcum, H. John Sharifi

**Affiliations:** Department of Biological and Environmental Sciences, Le Moyne College, Syracuse, NY 13214, USA

**Keywords:** HIV, Vpr, Vpx, CRL4, DCAF1, ubiquitin, G2 arrest, UNG2, SAMHD1, HuSH

## Abstract

The Human Immunodeficiency Virus (HIV) encodes several proteins that contort the host cell environment to promote viral replication and spread. This is often accomplished through the hijacking of cellular ubiquitin ligases. These reprogrammed complexes initiate or enhance the ubiquitination of cellular proteins that may otherwise act to restrain viral replication. Ubiquitination of target proteins may alter protein function or initiate proteasome-dependent destruction. HIV Viral Protein R (Vpr) and the related HIV-2 Viral Protein X (Vpx), engage the CRL4-DCAF1 ubiquitin ligase complex to target numerous cellular proteins. In this review we describe the CRL4-DCAF1 ubiquitin ligase complex and its interactions with HIV Vpr and Vpx. We additionally summarize the cellular proteins targeted by this association as well as the observed or hypothesized impact on HIV.

## 1. Introduction

### 1.1. Ubiquitination

Ubiquitin is an 8.5 kDa protein that is highly conserved and ubiquitously produced among all eukaryotes. Ubiquitination (also referred to as ubiquitylation) is a cellular process in which ubiquitin is covalently attached to target proteins. Ubiquitin can be attached singly at one lysine (K) residue of a target protein (mono-ubiquitination) or singly at multiple K residues of a target protein (multi-mono-ubiquitination). Ubiquitin can also be covalently linked to other ubiquitin proteins at K48 to form a ubiquitin chain that is covalently attached to a K residue on a target protein (poly-ubiquitination). Mono- and multi-mono-ubiquitination generally leads to altered protein function whereas K48-linked poly-ubiquitination typically labels a protein for degradation via the proteasome. The proteasome is a multimeric protein complex that hydrolyzes poly-ubiquitinated proteins into smaller peptides to be reused by the cell. Ubiquitination is extensively reviewed in [1].

The covalent attachment of ubiquitin to a protein is carried out by three classes of proteins: an E1 activating enzyme, an E2 conjugating enzyme, and an E3 ligase. The E1 enzyme uses ATP hydrolysis to form a thioester linkage with ubiquitin. The ubiquitin is then transferred to the E2 enzyme via a transthiolation reaction. The ubiquitin-charged E2 enzyme docks with the E3 ligase. Depending on the type of E3 ligase, the E3 ligase can either facilitate the direct transfer of ubiquitin from the E2 enzyme to the target substrate or act as a catalytic intermediate for the transfer. The E3 ligase determines the specific substrate(s) to be ubiquitinated. In humans, at least eight E1 enzymes, 40 E2 enzymes, and four broad classes of E3 ligases have been identified. E1 and E2 enzymes are extensively reviewed in [2] and [3], respectively. E3 ligases can consist of a single subunit or multiple subunits depending on the type of E3 ligase. E3 ligases are further categorized by the characteristic protein domain they carry: Really Interesting New Gene (RING) domain, Homologous to E6AP C-Terminus (HECT) domain, U-box domain, and RING-Between-RING (RBR) domain. Of these, RING domain containing E3 ligases are the most common with 600 different RING-type ligases identified in humans (reviewed in [4]).

### 1.2. Cullin-RING-E3 Ubiquitin Ligases (CRLs)

CRLs are a class of multi-subunit E3 ligases hallmarked by a cullin protein. In humans, eight cullin (Cul) proteins have been identified: Cul1-3, Cul4A, Cul4B, Cul5, Cul7, and Cul9 (PARC). The cullin protein acts as a scaffold to bridge two sets of functional subcomplexes. Substrate recognition complexes are engaged by the N-terminal end of cullins. Each cullin has α-helical bundles near its N-terminus. Some of these α-helices have unique amino acid residues that help to determine which substrate recognition complexes engage a particular cullin. Further, each CRL substrate recognition complex employs unique protein-protein interaction motifs to determine the pool of proteins to be targeted by that specific CRL (reviewed in [5]). For example, SKP1 engages unique residues in specific α-helices near the N-terminus of Cul1 [6]. SKP1 in turn engages any one of various possible substrate recognition proteins carrying an F box motif [7]. A particular F box motif-containing protein can then recruit specific substrate proteins through other unique motif interactions such as those facilitated by leucine-rich repeats [8]. 

While the N-terminal end of cullins determines substrate specificity, the C-terminal end engages a RING-domain containing protein that recruits the E2 conjugating enzyme and promotes its activity. This forms the catalytic subcomplex. At least two RING-domain-containing proteins have been identified that enable CRL function: RBX1 and RBX2 (also known as ROC1 and ROC2, respectively). RBX1 can serve all cullins except Cul9 (PARC), which is served by a yet-to-be-identified RING-domain-containing protein. RBX2 is predominantly associated with Cul5 [5].

Thus, each CRL is assigned a subset of proteins for ubiquitination that is dictated and facilitated by the type of cullin protein that defines it. 

### 1.3. Viruses and CRL Complexes

Ubiquitination is important for numerous cellular processes. Viruses are obligate intracellular parasites that rely on host cell processes to enable viral replication and spread. It is, therefore, unsurprising that several human viruses have been identified that usurp the process of ubiquitination to favor viral replication. Often, this involves the hijacking and subsequent re-directing of cellular ubiquitin ligase complexes.

Respiratory Syncytial Virus (RSV), Human Papilloma Virus (HPV), Hepatitis B Virus (HBV), Rotavirus, and the Human Immunodeficiency Virus (HIV), have all been demonstrated to commandeer CRLs to target cellular proteins for destruction. The NS1 protein of RSV engages the Cul2 containing CRL complex (CRL2) to promote STAT2 degradation [9], thereby inhibiting the induction of a potentially restrictive interferon (IFN) response. The E7 protein of HPV engages CRL2 to initiate the destruction of the Retina Blastoma (RB) protein [10], a tumor suppressor that blocks entry into S-phase. Loss of RB may therefore encourage viral DNA synthesis whilst inadvertently triggering the excessive cellular DNA synthesis that is characteristic of papilloma formation. The HBx protein of HBV engages CRL4 to mediate destruction of Smc5/6. Smc5/6 would otherwise act to inhibit transcription from the viral genome [11]. Of note, this is a similar strategy employed by HIV and is discussed in Section 3.5. Interestingly, Rotavirus NSP1 engages CRL3 to target β-TrCP, an F box motif-containing substrate-recognition protein of the CRL1 complex, for destruction [12]. Loss of β-TrCP may favor Rotavirus replication as CRL1-β-TrCP activity can promote the immune-stimulating NF-κB pathway (reviewed in [13]).

The HIV genome encodes several specialized proteins (often referred to as “accessory” or “auxiliary” proteins) that are not required for the fundamental structural and genomic replication of the virus but instead enable viral replication via antagonization of host intracellular and immunological defenses. Several of these specialized proteins act through CRLs. HIV-1 Vif engages a CRL5 ubiquitin ligase complex to target the cellular APOBEC3 family of proteins for polyubiquitination and subsequent proteasomal degradation. For example, failure to degrade APOBEC3G allows for its incorporation into new virions. Upon infection of the next cell, APOBEC3G exerts its cytidine deaminase activity to introduce guanine to adenine (G to A) mutations in the viral DNA. The hypermutated viral DNA is now ineffectual as a template for the generation of successive viruses and is prone to degradation. APOBEC3G functions and Vif antagonism are reviewed in [14]. 

Like Vif, HIV-1 Vpu antagonizes cellular defenses through CRL-mediated ubiquitination. HIV-1 Vpu engages the CRL1 (SCFβ-TrCP) complex. This interaction enables Vpu to target the cellular proteins tetherin (BST-2), CD4, and PSGL-1. As its name implies, tetherin (BST-2) tethers nascent virus to the infected cells, thereby reducing viral dissemination. As a receptor for HIV entry, loss of CD4 is presumed to contribute to viral spread by reducing re-adsorption. PSGL-1 gets incorporated into new virions and subsequently inhibits entry into uninfected cells. Vpu functions are reviewed in [15]. 

Thus, CRL-dependent Vif and Vpu functions act to clear the path for the essential viral replication processes. 

### 1.4. HIV Viral Protein R (Vpr) and Viral Protein X (Vpx)

HIV-1 Vpr is a 14 kDa protein that is abundantly incorporated into the virion through interactions with the p6 domain of the Gag structural protein precursor. Vpr is considered the most enigmatic of HIV’s specialized proteins. HIV-1 Vpr initiates global changes within the cell, and numerous phenotypes have been ascribed to it. HIV-1 Vpr induces G2 cell cycle arrest in dividing cells (e.g., CD4^+^ T-cells), enhances the infection of terminally differentiated (non-dividing) myeloid lineage cells (e.g., macrophages), transactivates the HIV-1 Long Terminal Repeat (LTR) to promote viral gene expression, activates the DNA damage response, initiates depletion of several DNA repair proteins, modulates host cell signaling pathways and the host immune response, contributes to nuclear import of viral DNA, and induces apoptosis (reviewed in [16,17,18,19,20,21,22] and discussed in subsequent sections). The contributions of many of these phenotypes to HIV pathology remain unclear with mixed evidence as to the impact these functions have on viral replication. For example, HIV-1 Vpr binds to the cellular DNA repair enzyme UNG2 and initiates its depletion. The role of UNG2 in HIV biology remains unclear with evidence supporting beneficial, detrimental, or neutral impacts on HIV (discussed in greater detail in Section 3.3). Similar discrepancies arise for other HIV-1 Vpr phenotypes and likely reflect differences in experimental approaches, or the cell types being tested. Which experimental approach best reflects relevance to human infection is difficult to discern. Nonetheless, HIV-1 Vpr is critical to HIV replication in vivo as its loss greatly diminishes HIV pathology [23,24].

HIV-2 encodes two HIV-1 Vpr-*like* proteins: HIV-2 Vpr and Vpx. The two viral proteins share approximately 50% and 25% amino acid similarity to HIV-1 Vpr, respectively, and likely emerged from a gene-duplication event of an HIV-1 Vpr-*like* precursor [25]. Like HIV-1 Vpr, HIV-2 Vpr and Vpx are incorporated into virions via interactions with the p6 domain of the Gag structural protein precursor. HIV-2 Vpr can trigger G2 cell cycle arrest, analogous to HIV-1 Vpr. In contrast, HIV-2 Vpr does not deplete UNG2 and lacks many of the phenotypes associated with HIV-1 Vpr (described in Section 3). HIV-2 Vpx markedly enhances myeloid lineage cell infection. This phenotype is primarily attributed to HIV-2 Vpx’s capacity to initiate degradation of the antiviral protein SAMHD1. HIV-2 Vpx also promotes expression from the provirus through degradation of members of the HuSH complex. SAMHD1, HuSH, and other targets of Vpx are discussed in greater detail in Section 4. Neither degradation of SAMHD1 nor counteracting the HuSH complex are functions performed by either HIV-1 Vpr or HIV-2 Vpr. Thus, despite the similarity and ancestry of these viral proteins, HIV-2 Vpr and Vpx appear to have evolved to carry out unique functions that differ from those of HIV-1 Vpr.

Regardless of their functionally different outcomes, all three of these viral proteins (HIV-1 Vpr, HIV-2 Vpr, and HIV-2 Vpx) require engagement of the CRL4 ubiquitin ligase complex, through the adaptor protein DCAF1, to establish most of their attributed phenotypes.

### 1.5. The Aim of This Work

In this review, we describe the CRL4-DCAF1 complex, the interaction of HIV Vpr and Vpx with the complex, the targets of this interaction identified to date, and the proposed consequences of this interaction to HIV replication. Of note, we will focus predominantly on HIV Vpr and Vpx as opposed to the Simian Immunodeficiency Virus (SIV) orthologs: SIV Vpr and Vpx. We will generally not discuss the subset of Vpr and Vpx functions wherein interaction with DCAF1 is dispensable, such as the degradation of the transcriptional co-regulator PHF13 [26] by HIV-1 Vpr or the inhibition of the STING [27] and NFκB [28] signaling pathways by HIV-2 Vpx.

## 2. The CRL4-DCAF1 E3 Ubiquitin Ligase Complex

### 2.1. Key Components of CRL4-DCAF1

Cul4 serves as the scaffold protein to bridge the functional subcomplexes of CRL4. In humans, Cul4 exists in two forms: Cul4A and Cul4B. The genes encoding Cul4A and Cul4B are located on chromosomes 13 and X, respectively, and likely arose from a gene-duplication event that precedes the emergence of mammals. Transcripts for both *cul4* types are highly expressed in human leukocytes. The two Cul4 types share 82% identity, with the notable difference being that Cul4B possesses an N-terminus that is 149 amino acids longer and harbors a nuclear localization signal (NLS). Presumably, the N-terminal NLS of Cul4B allows it to better fulfill CRL4-dependent roles in the nucleus whereas Cul4A can remain in the cytosol to carry out CRL4-dependent roles there. Cul4A and Cul4B are described and compared extensively in [29]. This segregation of the two Cul4 types was observed, to some extent, in terminally differentiated human monocyte-derived macrophages (hMDMs). However, the presence of the N-terminal NLS of Cul4B does not necessarily mean that CRL4B complexes are exclusively found in the nucleus. Indeed, both Cul4 types were shown to have similar subcellular distributions in activated primary T-cells with each being readily detected in both the cytosolic and nuclear compartments. HIV Vpr and Vpx can act through either Cul4A or Cul4B-containing CRL4 complexes [30].

The respective N-termini of both Cul4A and Cul4B engage DNA-Damage Binding Protein 1 (DDB1), which acts as the core adaptor for the substrate recognition complex. DDB1 consists of three sequential tryptophan-aspartate (WD)40 β-propeller domains, designated BPA, BPB, and BPC. BPB engages the N-terminus of Cul4. The BPA-BPC WD40 domains of DDB1 act as docking sites for a plethora of proteins carrying one or two WDx-arginine(R) signature motifs within a WD40 domain [5]. These proteins are termed DDB1-Cul4 Associated Factors, or DCAFs. At least 50 human DCAFs have been identified to date [31]. HIV Vpr and Vpx specifically engage DCAF1 to access CRL4 (described in Section 3.1 and Section 4.1, respectively). Of note, DCAF1 is also known as Vpr Binding Protein (VprBP) owing to this interaction. The N-terminus of DCAF1 consists of a long Armadillo (ARM) domain in which two subdomains are present: a casein kinase (CK-Like) domain and a Chromo domain. The CK-Like domain affords kinase activity to DCAF1. The Chromo domain is associated with chromatin remodeling. The ARM domain is followed by a shorter LisH domain that facilitates DCAF1 homodimerization. A Helix-Loop-Helix (HLH) motif is present after the LisH domain just prior to the WD40 domain. The HLH motif and WD40 domain engage with the BPA-BPC domains of DDB1 to facilitate docking. At the very C-terminus of DCAF1 is an Acidic domain which enables interaction with a subset of cellular substrates (reviewed in [32]). 

The C-termini of both Cul4A and Cul4B engage RBX1. RBX1 recruits the E2 conjugating enzyme to form the catalytic subcomplex. Several E2 enzymes have been identified that can associate with RING-domain-containing proteins like RBX1 and RBX2. Ube2R1 (Cdc34), for example, has been shown to engage RBX1 [33]. The exact E2 conjugating enzyme(s) that enable HIV Vpr and Vpx function through CRL4 have not yet been characterized.

The functionality of CRLs, including CRL4, is dependent on neddylation. NEDD8 is a ubiquitin-like protein that is covalently attached to a specific K residue within the Cullin Homology Domain (CHD). The CHD is a highly conserved region of the C-terminus of all cullin proteins. Similar to ubiquitination, the process of neddylation is carried out through a cascade of NEDD8-dedicated E1, E2, and E3 enzymes. Covalent attachment of NEDD8 to cullins activates CRLs in several ways: Neddylation induces conformational changes that encourage RBX1 to bind the E2 ubiquitin-conjugating enzyme and bring the RBX1-E2 complex closer to the target substrate. Neddylation also prevents recognition of cullins by cullin-sequestering proteins such as CAND1 that would otherwise act to prevent association of cullins with their respective RBX [34]. Accordingly, CRLs can be inactivated through de-neddylation or inhibition of neddylation via multiple mechanisms. Neddylation is extensively reviewed in [35]. Both Cul4A and Cul4B were shown to be almost exclusively in the neddylated (operational) form in the nucleus of activated primary T-cells [30]. Neddylation is required for HIV Vpr and Vpx functions through CRL4 as well as Vif-mediated APOBEC3G degradation through CRL5 [36,37,38].

CRL4-DCAF1 function is also dependent on quaternary structure. Two separate CRL4-DCAF1 complexes can link together via the LisH domain of their respective DCAF1s to form a dimer. This dimeric form of CRL4 exhibits better ubiquitin ligase activity than the monomeric form in vitro [39]. Additional biochemical and structural work by Mohamed et al. suggests that CRL4-DCAF1 complexes can switch between an inactive tetrameric form and an active dimeric form. Of note, binding of HIV-1 Vpr with its target substrate (i.e., UNG2) favored a switch to the active dimer form [40]. While those studies utilized CRL4A complexes, others have shown that Cul4B co-immunoprecipitates Cul4A, which suggests that the two Cul4 types may exist in a multimeric complex [41,42]. If so, understanding how these heterodimeric complexes intersect with HIV biology may provide additional insight into Vpr and Vpx functions.

### 2.2. Physiological Roles of CRL4-DCAF1

The CRL4 complex plays critical roles in cell cycle progression, DNA damage repair, chromatin remodeling, and metabolism. As such, numerous developmental and physiological processes are influenced by CRL4 function, including embryogenesis, hematopoiesis, and gametogenesis. In line with these functions, perturbation of CRL4 complex components is linked to embryonic lethality, sterility, several types of cancer, and intellectual disability, depending on which component(s) are compromised.

The sequence and structural similarity of the two Cul4 types impart considerable functional redundancy, which is reflected in the mutual substrates for the processes listed above. For example, proteins involved in DNA replication such as the replication factor MCM10, DNA repair such as UNG2, cell cycle regulation such as p21, chromatin remodeling such as PR-Set7/SET8, and metabolism such as TSC2 have all been shown to be substrates of Cul4A- and Cul4B-containing CRL4 complexes (reviewed in [29]). Additionally, several mouse-model studies suggest one Cul4 type can compensate for the other when the function of one of the two is lost during certain stages of development (reviewed in [29,43]). This is further demonstrated when the function of both Cul4 types is simultaneously lost, resulting in impaired cell proliferation, increased apoptosis, and embryonic lethality [44,45]. 

Despite their structural similarities and functional overlap, the two Cul4 types are not entirely interchangeable. Subcellular localization, tissue-specific expression, and temporal differences contribute to unique roles for each Cul4 type. Cul4A plays a larger role in nucleotide excision repair (NER) in response to certain types of genotoxic stress such as ultraviolet irradiation. Cul4A is also critical for the resolution of double-stranded DNA breaks (DSBs) that occur during the meiotic recombination and crossover events of spermatogenesis. Male mice lacking *cul4a* are infertile as a result. The contribution of Cul4A to spermatogenesis likely explains why no humans have been identified as lacking functional Cul4A. The role of Cul4A in DNA repair is extensively reviewed in [46]. Human males lacking Cul4B, however, present with X-linked intellectual disability (XLID) accompanied by reduced motor coordination, irregular gait, and anatomical abnormalities, such as short stature and kyphosis (reviewed in [47]). Failure of CRL4B to specifically target the H3K4 methyltransferase component WDR5 leads to altered neuronal gene expression profiles and reduced neurite growth and may contribute to the development of XLID [48]. These unique phenotypes highlight the distinct contribution of each respective Cul4 type. 

As described in Section 2.1, DDB1 is the core adaptor for the CRL4 substrate recognition complex, and this is highlighted in mouse work wherein elimination or depletion of DDB1 phenotypically mimics the simultaneous loss of both Cul4A and Cul4B function [44]. This is also reflected in cell culture studies investigating Vpr and Vpx function [30,49]. DDB1, in complex with DDB2, scans the DNA for distortions such as UV-induced pyrimidine dimers. Upon detection of damage, Cul4A is recruited to the DDB1-DDB2 complex to initiate the ubiquitination of several proteins involved in NER and orchestrates both the repair process and the resolution of the response upon completion of the repair (reviewed in [46,50]). Of note, DDB1 is detectable in both the cytosolic and nuclear compartments in primary immune cell targets of HIV [30]. 

PIKES (Protein Interaction Kinetics and Estimation of Stoichiometries) analysis suggests DCAF1 is the second most common DCAF protein to be in complex with CRL4 [51]. DCAF1 is produced in several tissues, and loss of DCAF1 in mice is embryonic lethal [52]. Concordantly, DCAF1 has been found to have numerous roles in several physiological processes, including cell division, cytoskeletal organization, gametogenesis, myogenesis, microRNA (miRNA) biogenesis, and immune cell maturation and activation (reviewed in [32]). In the context of CRL4 function, DCAF1 activates members of the TET demethylase family to coordinate gene expression during oocyte maturation [53]. CRL4-DCAF1 may also contribute to lipid metabolism via downregulation of the nuclear receptor TR4, a process that may be inhibited by SIRT7 [54]. Further, CRL4-DCAF1 can modulate the levels of the endoribonuclease Dicer in a tissue-specific manner [52,55]. Dicer plays a key role in miRNA processing. miRNAs contribute to post-transcriptional gene silencing by acting to repress translation of target messenger RNAs (mRNA).

Interestingly, a subset of DCAF1 functions appear to be independent of its association with CRL4. The Chromo domain of DCAF1 is a domain architecture commonly found in proteins involved in chromatin remodeling and gene regulation. This suggests that DCAF1 can physically interact with chromatin to influence gene expression. Indeed, DCAF1 can directly bind to histone H3 tails at p53 responsive genes to block transcription. Though this was mapped to DCAF1′s LisH domain, it nonetheless highlights a direct role for DCAF1 in influencing gene expression [56]. Of note, DCAF1′s influence on p53 contributes to a vital role in activated CD4^+^ T-cell growth and cell cycle entry [57].

p53 is best known for its function as a tumor suppressor. In addition to negatively modulating p53 activity, DCAF1 can also impair the function of other tumor suppressors. This is accomplished through an intrinsic kinase activity mediated by the CK-Like domain. Through its CK-Like domain, DCAF1 phosphorylates histone H2A at threonine 120 (T120). H2AT120 phosphorylation is connected to repression of tumor suppressor genes [58]. Thus, DCAF1 can exert its kinase activity to influence cell cycle regulation via chromatin remodeling. Future work is likely to reveal a broader role for DCAF1′s intrinsic kinase activity.

**Figure 1:**
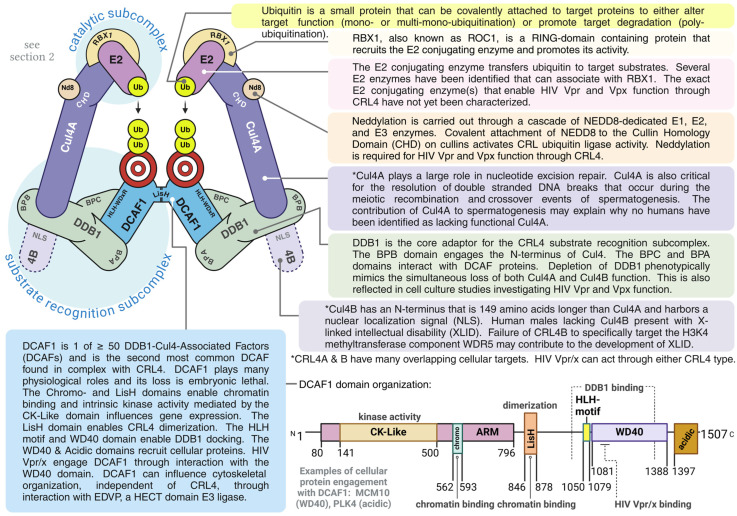
Overview of the CRL4-DCAF1 Ubiquitin Ligase Complex. Created with BioRender.com.

DCAF1 can further influence cell cycle progression via its contribution to cytoskeletal reorganization, specifically affecting centrosome structure and function. The centrosome is responsible for organizing the microtubules that form the mitotic spindle. DCAF1 is implicated in the appropriately timed turnover of Katanin and CP110 to ensure proper segregation of daughter chromatids. Importantly, this function is attributed to DCAF1 interaction with EDVP, a HECT domain E3 ligase, and does not appear to overlap with CRL4-DCAF1 functions [59]. The structural and physiological characteristics of CRL4-DCAF1 are displayed in Figure 1.

In alignment with the nature of viruses, several of the aforementioned physiological targets, processes, and interactions are directly manipulated by HIV Vpr and Vpx and are discussed, in that context, in the subsequent sections.

## 3. CRL4-DCAF1 and HIV Viral Protein R

In the following subsections we discuss Vpr’s interaction with CRL4-DCAF1 and the cellular targets of this interaction. For brevity, certain experimental details have been excluded; however, much of the work described relied on commonly accepted approaches for the determination of Vpr function through CRL4. Examples of these approaches include, but are not limited to, co-immunoprecipitation assays, RNAi-mediated depletion of CRL4-DCAF1 components, chemical inhibition of proteasomal (e.g., MG132) or CRL4 function (e.g., inhibition of neddylation via MLN4942), or the use of Vprs harboring alterations that have well-established functional outcomes (e.g., Q65R impairing DCAF1 binding or R80A abrogating G2 cell cycle arrest, discussed below).

Given the numerous phenotypes associated with Vpr, and the array of cellular processes regulated by CRL4-DCAF1, it is inevitable that targets predominantly associated with one Vpr phenotype will have overlapping contributions to another Vpr phenotype. Such overlaps will be noted and described. 

### 3.1. Vpr, CRL4-DCAF1, and Target Protein Interactions

HIV-1 Vpr consists of 96 amino acids that form an unstructured N-terminal tail, followed by three α-helices, mapping to amino acid positions 17–33, 38–50, and 55–77, respectively, and an unstructured C-terminal tail. Vpr contains four structural motifs: the N-terminal tail; a hydrophobic cleft between α-helix 1, 2, and the first turn of α-helix 3; the insert loop connecting α-helix 2 and α-helix 3; and the C-terminal region of α-helix 3. α-helix 3 and the random coil N-terminal tail of Vpr are employed to engage DCAF1. α-helix 3 of Vpr interacts with a cleft on the surface of DCAF1 through a mixture of hydrophobic interactions and hydrogen bonding; specifically, a crystal structure showed residues R62, Q65, and R73 on α-helix 3 of Vpr forming hydrogen bonds with DCAF1. Residues E25, L26, and E29 on α-helix 1 coordinate with Q65 and L68 on α-helix 3 to form a small pocket where DCAF1 residue W1156 binds. The N-terminal tail of Vpr can then wrap around DCAF1. Consistent with these findings, Vpr R62D fails to effectively bind with DCAF1. Vpr F69A also fails to bind DCAF1 owing to disruption of hydrophobic interactions between Vpr and DCAF1 residues [60]. Furthermore, a highly conserved Wx4Φx2Φx3AΦxH motif is present within α-helix 1 of HIV-1 Vpr, HIV-2 Vpr, and HIV-2 Vpx. In HIV-1 Vpr, the Wx4Φx2Φx3AΦxH motif maps to residues 18–33 in α-helix 1 and is required for Vpr-DCAF1 interactions and Vpr-induced G2 cell cycle arrest. This is supported with Vpr double alterations: L22S/L23S and A30S/V31S, which both exhibit reduced DCAF1 binding and impaired induction of G2 cell cycle arrest [61]. 

Other work identified the presence of a highly conserved HHCH/HHCC motif. This motif maps to amino acid positions 33, 71, 76, and 78 and is critical for Vpr-CRL4 assembly. This is supported by mutagenetic experiments wherein HIV-1 Vpr H33A, H71A, C76S, and H78A alterations each demonstrated impaired DCAF1 binding, attenuated G2 cell cycle arrest induction, and reduced HLTF (discussed below) degradation. Treatment with TPEN, a zinc chelator, yielded similar results highlighting the importance of zinc-binding to the function facilitated by this highly conserved motif [62]. 

Notably, Vpr residue Q65 plays a larger role in the Vpr-DCAF1 interaction and influencing how Vpr accumulates in the cell. HIV-1 Vpr primarily localizes in the nucleus, but also assembles at the nuclear envelope. Vpr Q65R alteration failed to accumulate at the nuclear envelope. This indicates that interaction with DCAF1 is necessary for proper localization of Vpr at the nuclear envelope, a phenotype that may contribute to reduced IRF3 and NF-κB immune activation (discussed in Section 3.5). Thus, the single change of Vpr Q65R abolishes the ability of Vpr to engage DCAF1, to accumulate at the nuclear envelope, and to induce G2 cell cycle arrest [63]. These results underscore the importance of Vpr’s interaction with DCAF1 to the viral protein’s functions through CRL4. 

Vpr binding to DCAF1 is necessary, but not sufficient, to trigger G2 cell cycle arrest. Residue R90 is not involved in DCAF1 binding, and the alterations R90K and R90D both interact with DCAF1. However, Vpr R90D can both induce G2 cell cycle arrest and degrade UNG2, while R90K cannot perform either function. Importantly, Vpr R90K weakly associates with DDB1, while Vpr R90D demonstrates wild-type-*like* association with DDB1. This indicates that Vpr must associate with both DCAF1 and DDB1 in order to effectively function and that binding of Vpr to DCAF1 alone does not ensure access to DDB1. These findings highlight that additional interactions or modifications are likely required for Vpr-DCAF1 to engage with DDB1 [64].

The C-terminal region of α-helix 3 and the C-terminal tail of Vpr are crucial for inducing Vpr-mediated G2 cell cycle arrest but do not affect the ability of Vpr to engage DCAF1. This is evident by removal of the C-terminal tail of Vpr, which did not affect binding to DCAF1 but abrogated the ability of Vpr to induce G2 cell cycle arrest. This observation led to the discovery of a conserved SRIG motif (residues 79–82) within the C-terminal tail of both HIV-1 and HIV-2 Vpr. The SRIG motif is not present on HIV-2 Vpx, which does not induce cell cycle arrest. The SRIG motif is essential for Vpr-mediated G2 cell cycle arrest. Using site-directed mutagenesis, Vpr alterations were created by substituting alanine for each residue of the SRIG motif (S79A, R80A, I81A, and G82A). All four Vpr alterations were unable to induce G2 cell cycle arrest [65]. The importance of the SRIG motif for Vpr-mediated G2 cell cycle arrest is further supported by Vpr R80A, which cannot cause G2 cell cycle arrest yet maintains the ability to interact with DCAF1 and exerts a dominant negative effect by competing with wild-type Vpr for DCAF1 binding [66]. These findings emphasize R80’s role in engaging the G2 cell cycle arrest target(s). The C-terminal SRIG motif, however, does not act alone in facilitating G2 cell cycle arrest. Vpr K27M and Vpr K27M/S79A alterations both successfully recruit DCAF1 yet fail to induce G2 cell cycle arrest. Therefore, the C-terminal tail of Vpr is not the only factor contributing to the induction of G2 cell cycle arrest. It is likely that non-linear determinants involving the core helical domain of Vpr coordinate with the C-terminal tail to effectively recruit the G2 cell cycle arrest target(s) [65,67]. Targets of Vpr associated with G2 cell cycle arrest are discussed in Section 3.2. 

Interestingly, comparison between Vpr S79A and Q65R revealed that, while both did not induce G2 cell cycle arrest, Vpr S79A led to cytotoxic phenotypes characteristic of apoptosis whereas Vpr Q65R did not. Unlike Vpr Q65R, Vpr S79A retains its association with DCAF1 [65]. These results suggested that HIV-1 Vpr-mediated cell death may not necessarily be due to prolonged arrest in G2 but might instead be due to targeting of a distinct cellular protein via DCAF1. A potential target for this effect is described in Section 3.6.

HIV-1 Vpr binds to the targets UNG2 and HLTF through its core helical domain (α-helices 1–3), opposite to Vpr’s interface with DCAF1. The insert loop of Vpr engages UNG2 as the residues forming the hydrophobic cleft lock in the binding of Vpr to UNG2. For example, hydrogen bonds form between D52 of Vpr and Y147 of UNG2 while stacking interactions form between W54 of Vpr and P168 of UNG2 [60]. Consistent with this finding, Vpr W54R and W54G alterations fail to bind [68], and subsequently downregulate [69], UNG2. The insert loop of Vpr mimics DNA for recognition of UNG2’s DNA binding site, allowing Vpr residue D52 to directly interact with the UNG2 DNA binding site, while α-helix 3 of Vpr forms close contacts with the residues of UNG2 that intercalate into the DNA helix when UNG2 binds DNA. This suggests that Vpr utilizes molecular mimicry of DNA for specific recruitment of UNG2 [60]. 

**Figure 2:**
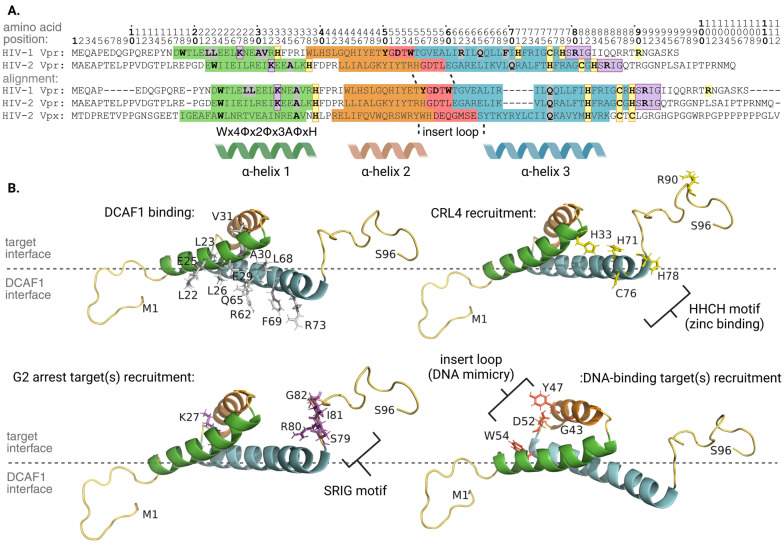
Interaction properties of HIV-1 Vpr. (**A**) Amino acid sequences of HIV-1 Vpr from HIV-1 89.6 (accension Q73369) and HIV-2 Vpr and Vpx from HIV-2 ROD (accension P06938 and P06939, respectively). (**B**) Ribbon and stick representations of HIV-1 Vpr (from PDB 1M8L). Key residues, motifs, and domains for the indicated interactions are color coded and/or bolded and/or labeled for each panel. Ribbon and stick representations were created with the PyMOL Molecular Graphics System, Version 2.5.5. This figure was assembled with BioRender.com.

This same DNA mimicry is employed by HIV-1 Vpr to mediate the recruitment and degradation of another DNA repair protein, HLTF. Unlike UNG2, however, two separate interactions of Vpr with HLTF facilitate polyubiquitination and degradation. In brief, HLTF consists of a DNA-binding HIRAN domain, a LINKER region, and a helicase domain. The HIRAN domain and LINKER region constitute the N-terminal domain (NTD). Vpr shows affinity for both the HIRAN domain and, separately, the LINKER region of HLTF. Mutagenic analysis revealed that HIV-1 Vpr D52A, present within the DNA mimicking insert loop, fails to mediate polyubiquitination of the HLTF-HIRAN domain, but does not influence polyubiquitination of the HLTF-LINKER region. Vpr G43W/Y47V double alteration, however, failed to mediate polyubiquitination of the HLTF-HIRAN domain and exhibited reduced polyubiquitination of the HLTF-LINKER region. This latter observation was associated with a fourfold reduction of the HLTF-NTD binding to DCAF1-Vpr. Together, these data support a model wherein Vpr residue D52 exclusively interacts with the HIRAN domain of HLTF while residues G43 and Y47 interface with both the HIRAN domain and the LINKER region. Thus, HIV-1 Vpr uses molecular mimicry of DNA to recruit DNA repair proteins to DCAF1, but the nature and number of interactions are likely target-specific [70]. The interaction properties of HIV-1 Vpr are presented in Figure 2.

The interplay between DNA repair protein targets and HIV biology is discussed in Section 3.3.

### 3.2. Targets Associated with G2 Cell Cycle Arrest

One of the first phenotypes attributed to Vpr was its ability to arrest cycling cells in the G2/M phase of the cell cycle. This was observed in both cell lines and in CD4^+^ T-cells from infected patients, indicative of biological relevance [71,72]. Vpr can trigger cell cycle arrest when introduced in isolation or in the context of infection, and this phenotype is conserved between HIV-1 Vpr and its paralog HIV-2 Vpr. Vpr-arrested cells exhibited 4C DNA content revealing a block at either the G2 or M phase of the cell cycle. Vpr was found to block activation of the p34cdc2/cyclin B complex, thereby pinpointing the arrest phenotype to the G2 phase [73,74], although the events that initiate G2 arrest may occur during S phase [75]. Vpr is dispensable for HIV replication in most dividing cell lines and has a modest impact on infection of cycling primary CD4^+^ T-cells in culture [76]. These observations made it challenging to understand the contribution of G2 cell cycle arrest to pathology; however, a cell in the G2 phase is undergoing robust transcription and translation in preparation for mitosis. Thus, the cellular environment of a G2-arrested cell is likely most conducive to HIV gene expression and viral protein production. Indeed, up to a threefold increase in viral production has been observed when cells are arrested in the G2 phase as compared to virus production from an asynchronous population [77]. 

The mechanism by which Vpr induces cell cycle arrest came to light after a burst of publications identified the CRL4-DCAF1 ubiquitin ligase complex as an interaction partner for Vpr [49,66,78,79,80,81,82]. CRL4 activity, K48-linked polyubiquitination, and proteasomal function were all confirmed necessary for Vpr to cause G2 cell cycle arrest [83]. Upon these findings, it was anticipated that identification of the cellular protein targeted for degradation by Vpr would, like for Vif and Vpu, provide a clearly defined biological relevance. However, the cellular protein(s) that Vpr targets to induce G2 cell cycle arrest have proven difficult to pin down and are the subject of much contention. 

SLX4 is a scaffold protein that recruits structure-specific endonucleases, such as SLX1, MUS81-EME1, and ERCC1-ERCC4XPF, to form the SLX4 complex (SLX4com). SLX4com repairs DSBs, interstrand cross-links, and collapsed or damaged replication forks caused by homologous recombination. The function of the structure-specific endonucleases is to remove branched or multistranded DNA structures that are created during DNA replication and repair processes (reviewed in [84]). MUS81-EME1 also fixes collapsed replication forks by removing ultra-fine DNA bridges (UFBs) that form between sister chromatids in slower-replicating genomic regions (reviewed in [85]). Activation of MUS81-EME1 is restricted to the late G2/early M phase of the cell cycle after most of the DNA synthesis has occurred such that healthy replication forks are not destroyed during S phase [86]. In mammalian cells, MUS81-EME1 activity is regulated by the phosphorylation of EME1 inside SLX4com. The phosphorylation of EME1 is associated with increased cleavage activity of MUS81 [87].

Laguette et al. identified an interaction between SLX4com and HIV-1 Vpr that may play a part in Vpr-mediated G2 cell cycle arrest. Vpr binds to the C-terminus of SLX4, recruiting DCAF1 and kinase-active polo-like kinase 1 (pPLK1). Then, with the assistance of DCAF1, MUS81 is ubiquitinated and EME1 is hyperphosphorylated. This prematurely activates MUS81-EME1 outside of the G2/M phase of the cell cycle [88] and is consistent with Vpr triggering events in S phase that lead to arrest in G2 [75]. Repeated activation of MUS81-EME1 leads to replication stress as the endonucleases break down DNA at improper times in the cell cycle and process replication forks in a dysfunctional manner. The resultant DSBs induce the ATR pathway, possibly leading to G2 cell cycle arrest. Of note, SLX4 may be an ATR substrate [89,90]. Activation of ATR by HIV-1 Vpr is discussed in greater detail in Section 3.3. SLX4 is also known as FANCP and is a part of the Fanconi Anemia DNA repair pathway alongside 15 other proteins. FANCM binds chromatin at sites of DNA damage. Upon binding, the FA core complex, which harbors E3 ubiquitin ligase activity, is recruited to the site of DNA breakage and monoubiquitinates Fanconi Anemia Group D2 protein (FANCD2)-FANCI to stabilize these proteins at the site of DNA damage. FANCD2-FANCI then activates DNA repair proteins such as SLX4com [84]. To this end, the presence of Vpr increased FANCD2 foci. Typically, high levels of FANCD2 foci indicate replication stress and unresolved replication intermediates. The increase in endonuclease activity correlating with an increased amount of FANCD2 foci is suggestive of DNA damage accumulating due to complications with the cleavage of replication intermediates. Thus, the inappropriate activation of SLX4com by Vpr could initiate the cascade of events that trigger G2 cell cycle arrest, and this is supported by Vpr interacting with SLX4 prior to the onset of the arrest. Further, RNAi-mediated depletion of the SLX4com components SLX4, MUS81, or EME1 decreased Vpr’s capacity to induce G2 cell cycle arrest [88].

Having established a plausible mechanism for the induction of G2 cell cycle arrest by HIV-1 Vpr, Laguette et al. set out to investigate the biological relevance of their observations. In the process of converting the HIV genome from single-stranded RNA to double-stranded DNA (dsDNA), many intermediate forms of nucleic acid can form. These intermediate nucleic acid forms can be detected by nucleic acid sensors present in the host cell, leading to the induction of a signaling cascade and the production of proinflammatory cytokines. Additionally, up to 90% of completed HIV-1 reverse transcripts do not integrate (reviewed in [91]), potentially allowing for these viral DNA products to signal to the cell that an infection has occurred. Therefore, the virus would benefit from removing these intermediate and unintegrated forms of nucleic acid such that they do not trigger an immune response. HIV-1 Vpr may therefore target SLX4com to avoid triggering the innate immune response. Indeed, cells depleted of SLX4 components demonstrated increased IFN induction in response to single round reporter HIV-1 transduction. These data suggested that HIV-1 Vpr recruitment of SLX4 and activation of MUS81-EME1 is a method for removing excess viral DNA to impede type 1 IFN production. As such, transduction with a Vpr-null virus increased types I IFNα and IFNβ expression relative to HIV-1 and SLX4 was demonstrated to be recruited to HIV DNA during reverse transcription. Additionally, RNAi silencing of SLX4 led to the accumulation of viral DNA. Together, these findings favored the hypothesis that MUS81-EME1 endonucleases remove excess reverse-transcribed HIV DNA to prevent innate immune sensing. If SLX4 helps HIV-1 to evade the immune response, then loss of SLX4 should impede HIV-1 infection. This expected decrease in HIV-1 infection was observed in cells collected from FANCP patients wherein SLX4 is absent. Interestingly, MUS81 ubiquitination was increased by Vpr, and this corresponded to a loss of MUS81. The reduction of MUS81 is counterintuitive as it would be expected to compromise SLX4com function, but reduction was observed mostly after Vpr’s activation of MUS81-EME1 via pPLK1 [88]. It was proposed that loss of MUS81 impairs UFB resolution and contributes to G2 arrest, but this was later demonstrated not to be the case (discussed below). 

Berger et al. confirmed SLX4com as a binding partner of HIV-1 Vpr and found SLX4com to be a common binding partner of many related SIV Vprs. This finding implied interaction with SLX4com is a conserved function of Vpr. Concordantly, all SIV Vpr alleles that caused G2 cell cycle arrest in human cells also interacted with SLX4com in a manner that requires engagement of DCAF1. These data seemingly solidified the link between Vpr, SLX4com, and G2 cell cycle arrest. However, HIV-1 Vpr R80A, which cannot cause G2 cell cycle arrest but retains interaction with DCAF1 (discussed in Section 3.1), associated with both DCAF1 and SLX4, thereby weakening the link of this association with G2 cell cycle arrest [92].

A conserved function of human and primate lentivirus Vprs is the ability to induce G2 cell cycle arrest in dividing cells. Therefore, if this phenotype is presumed to act through a singular mechanism, that mechanism should also be conserved. While work by Berger et al. demonstrated SLX4com engagement to be conserved by many SIV Vprs, Fregoso and Emerman provided evidence that SLX4com activation was not universally correlated with the induction of G2 cell cycle arrest. It was determined that the interaction of Vpr with SLX4 was not conserved across all HIV-1 and HIV-2 isolates. Since HIV-1 and HIV-2 followed different evolutionary paths and both can infect human cells, having a shared interaction of their respective Vprs with SLX4 would demonstrate that the interaction is an important part of Vpr function. However, the lack of conservation suggests that engagement of SLX4com is not a universal requirement for Vpr to induce G2 cell cycle arrest. Further, CRISPR-Cas9-mediated knockout of SLX4 did not restrict the ability of Vpr to activate the DNA damage response or arrest the cell cycle. The authors postulated that the observed interaction of some Vprs with SLX4 may instead be due to the recruitment and degradation of MUS81. SLX4 was not needed for degradation of MUS81 by Vpr, and MUS81 degradation was not correlated with G2 cell cycle arrest. This work concluded that SLX4 is not necessarily required for Vpr-mediated G2 cell cycle arrest [93]. 

Zhou et al. further determined that the downregulation of MUS81 by HIV-1 Vpr can occur independently of an interaction with SLX4. Vpr was shown to down-regulate levels of both MUS81 and its partner EME1 via CRL4-DCAF1-mediated degradation. Unlike the association with SLX4, down-regulation of MUS81-EME1 is evolutionarily conserved among Vprs, implying that the activity of MUS81-EME1 must be detrimental to HIV-1 replication. As was observed for the work described above, down-regulation of MUS81-EME1 was not correlated to the induction of G2 cell cycle arrest as several alterations of Vpr that impair G2 cell cycle arrest could still facilitate reduction of MUS81-EME1 levels. Interestingly, Vpr Q65R, which is impaired in DCAF1 binding, could still facilitate MUS81 degradation. The authors surmised MUS81 recruitment must facilitate a conformational change that compensates for an otherwise reduced ability of Vpr Q65R to engage DCAF1 [94]. Of note, the key experiments in this work were performed in the HEK293T cell line whereas the key experiments in the work by Laguette et al. were performed in the HeLa cell line. It is therefore possible that the discrepancies observed among the publications discussed arise from inherent differences in experimental systems. The benefit of MUS81 down-regulation to HIV infection has yet to be determined. SLX4com and HIV are reviewed in [95,96,97,98].

In all, the findings discussed above posit that Vpr can target other cellular proteins to induce G2 cell cycle arrest. As discussed in Section 2.2, MiniChromosome Maintenance complex component 10 (MCM10) is a native substrate for CRL4-DCAF1. MCM10 binds to the origin of replication in eukaryotic genomes to help stabilize DNA polymerase-α and promote the initiation and elongation steps of DNA replication. Nonfunctional MCM10 can cause replication fork stalling, leading to cell cycle arrest in late S phase (reviewed in [99]). These two observations led to the hypothesis that Vpr may enhance MCM10 degradation to possibly contribute to G2 cell cycle arrest by HIV-1 Vpr. Indeed, HIV-1 Vpr can bind to and deplete MCM10 directly on chromatin, making this the first study to demonstrate that an HIV-1 accessory protein can directly target a cellular protein on chromatin. Depletion of MCM10 by HIV-1 Vpr required interaction with DCAF1 and a functional proteasome. Importantly, RNAi-mediated depletion of MCM10 caused the accumulation of cells in the G2 phase of the cell cycle, and exogenous complementation of MCM10 in the presence of Vpr inhibited Vpr-mediated G2 cell cycle arrest in a dose-dependent manner [100]. Subsequent work found HIV-1 Vpr alterations that could not induce G2 arrest were also incapable of degrading MCM10, thereby strengthening the link between these phenotypes. However, MCM10 degradation was not conserved across Vprs from various lineages, suggesting that MCM10 degradation may not be a universal contributor to Vpr-mediated G2 cell cycle arrest [101]. Of note, while these studies utilized non-immune cell lines to carry out their respective experiments, other work connected MCM10 loss to increased G2 cell cycle arrest in the CEM-T4 CD4^+^ T-cell line [102].

Work by Zhang and Bieniasz identified CCDC137 (cPERP-B) as a target of HIV-1 Vpr. Using a proximity-dependent method, in the presence of proteasome inhibitor, CCDC137 was identified as an interaction partner of HIV-1 Vpr. In brief, Vpr is joined with an enzyme capable of biotinylating proteins. This enzyme-fused Vpr is then used as bait. Other cellular proteins in the vicinity of the bait are biotinylated. The biotin tag can then be used to isolate and identify interaction partners of the bait (detailed in [103]). HIV-1 Vpr and, to a lesser extent, HIV-2 Vpr both depleted CCDC137 in multiple cell types, including primary immune cells. Association with DCAF1 was demonstrated to be required for CCDC137 depletion by Vpr. Introduction of CCDC137 alterations that could not be degraded by Vpr blocked G2 cell cycle arrest in the presence of Vpr. Further, RNAi-mediated CCDC137 depletion alone was sufficient to cause accumulation of cells in the G2 phase of the cell cycle and form γ-H2AX foci, another phenotype attributed to Vpr. Importantly, CCDC137 depletion increased HIV-1 gene expression in both hMDMs and primary CD4^+^ T-cells, yet another phenotype ascribed to Vpr. Additionally, the enhanced HIV gene transcription upon reduction of CCDC137 was greater in hMDMs [104], where HIV-1 Vpr exerts a more obvious benefit to HIV-1 replication (discussed in Section 3.4). Interestingly, the function of CCDC137 is largely unknown. CCDC137 has been shown to trap retinoic acid receptor in the nucleolus [105], but there is no clear relevance of that action to HIV gene expression. Nonetheless, the attraction of CCDC137 is that it unifies several phenotypes ascribed to HIV-1 Vpr.

SLX4com, MCM10, and CCDC137 represent a fraction of the total proteins that may contribute to Vpr-mediated G2 cell cycle arrest. In the subsequent sections, targets associated with other Vpr functions that may also contribute to G2 cell cycle arrest will be described. Additionally, G2 cell cycle arrest by Vpr will be revisited in Section 5.3.

### 3.3. Targets Associated with the DNA Damage Response/DNA Repair

G2 cell cycle arrest is often a cellular response to DNA damage. HIV-1 Vpr localizes to the nucleus of cells [106,107,108]. These two observations led to the hypothesis that Vpr may activate the DNA damage response. Two proteins involved in detecting damaged DNA are Ataxia-Telangiectasia-Mutated kinase (ATM) and ATM and Rad3-related kinase (ATR). Vpr was found to activate ATR as indicated by Chk1 activation, which would presumably initiate a signaling cascade that prevents the cell from moving past the G2/M checkpoint [109]. How Vpr activates the ATR pathway is incompletely understood. Vpr may induce DSBs in the DNA or initiate the degradation of a protein involved in modulating the DNA damage response, or some combination of both. K48-linked polyubiquitination, proteasomal degradation, and Vpr’s ability to engage CRL4-DCAF1 are connected to Vpr activating the ATR response [83]. Additionally, Vpr unwinds dsDNA to recruit RPA70, a subunit of RPA that activates the ATR response. Importantly, this required Vpr-mediated ubiquitination of histone H2B, and Vpr Q65R failed to promote H2B ubiquitination, implying a role for CRL4-DCAF1. Further, in the Mit-23 cell line, dsDNA unwinding by Vpr created negative supercoiling leading to DSBs as a consequence of topoisomerase I function. While RNAi-mediated topoisomerase I depletion slightly reduced the ability of HIV-1 Vpr to cause G2 cell cycle arrest, this was not statistically significant. However, in hMDMs, depletion of topoisomerase I suppressed viral DNA integration. Thus, the influence of Vpr on DNA structure may enable efficient integration of viral DNA [110]. Other targets associated with HIV-1 Vpr-mediated enhancement of macrophage infection are discussed in Section 3.4.

Uracil-DNA-Glycosylase 2 (UNG2) and Single-strand-selective Monofunctional Uracil-DNA-Glycosylase 1 (SMUG1) were the first identified cellular targets of CRL4-DCAF1-dependent degradation by HIV-1 Vpr [69,80,111,112]. Interestingly, the majority of subsequent studies focused on UNG2 rather than SMUG1 possibly on the assumption that results obtained for UNG2 would hold true for SMUG1. As alluded to in Section 1.4, the role of UNG2 in HIV biology is unclear with evidence to support beneficial, detrimental, and neutral effects on viral replication (reviewed in [113]). For example, poor fidelity of the HIV reverse transcriptase enzyme can lead to the misincorporation of uracil into the viral DNA. UNG2 is a cellular DNA repair protein that contributes to the base excision repair pathway by excising misincorporated uracil, allowing for the eventual incorporation of the correct base. Thus, HIV-1 Vpr-mediated inclusion of UNG2 into the virion can help to increase the fidelity of reverse transcription, and several studies provide evidence in support of this beneficial role [111,114,115,116,117]. However, other work found UNG2 to aid in the degradation of viral DNA edited by APOBEC3G [118], and some level of viral DNA uracilation appears to prevent its autointegration and subsequent degradation [119]. Thus, HIV-1 Vpr-mediated UNG2 depletion would act to counter a detrimental role for UNG2. Additionally, HIV-1 Vpr can suppress *ung2* expression through promoter repression [120]. The fact that HIV-1 Vpr employs two mechanisms to reduce UNG2 levels strongly suggests that, despite a potentially beneficial role, UNG2 plays an overall inhibitory role, though the exact nature of such a detrimental role remains elusive. However, other work found UNG2 to have no effect on HIV replication in either CD4^+^ T-cell lines or hMDMs [121]. Further complicating our understanding of UNG2 in HIV biology is that the loss of UNG2 is not linked to Vpr-mediated G2 cell cycle arrest [68], and UNG2 depletion is not a trait associated with HIV-2 Vpr or several SIV Vprs [122]. Regardless, the study of UNG2 has proven invaluable by providing insight into how HIV-1 Vpr engages CRL4-DCAF1 to promote ubiquitination of cellular targets (described in Section 3.1).

After the identification of UNG2 as a CRL4-DCAF1-dependent target of HIV-1 Vpr, Wen et al. demonstrated that UNG2 and SMUG1 are normal substrates of the CRL4-DCAF1 complex and that HIV-1 Vpr acts to enhance this constitutive turnover [123]. This initial finding set forth the possibility that HIV-1 Vpr engaging CRL4-DCAF1 may enable it to access a bevy of normal physiological substrates of CRL4-DCAF1 that might otherwise act to impede viral replication. While not all proteins targeted by this interaction have been shown to be normal physiological substrates of CRL4-DCAF1, several have. Not surprisingly, many of these targets are involved in cellular processes in which CRL4-DCAF1 is involved, such as cell cycle progression, DNA repair, histone modification, and others (described in Section 2.2, Section 3.2, and below).

Helicase-Like-Transcription Factor (HLTF) is involved in several DNA repair processes, such as DNA replication fork regression in response to genotoxic stress (reviewed in [124]). Like UNG2, HLTF is degraded by HIV-1 Vpr through DCAF1, in a proteasome-dependent manner. HLTF degradation is also not linked to G2 cell cycle arrest. Unlike UNG2, HLTF does not appear to be a normal substrate of CRL4-DCAF1 in immune cells, as DCAF1 depletion or proteasome inhibition did not affect basal HLTF levels in uninfected cells. HLTF is degraded by virion-associated HIV-1 Vpr early after infection in HeLa cells, the CD4^+^ T-cell lines: Jurkat and MT4 and in hMDMs. HIV-1 Vpr did not enhance infection of these cell types despite HLTF degradation. Given HIV-1 Vpr’s noticeable impact on enhancing hMDM infection, RNAi-mediated depletion of HTLF was tested in hMDMs but reducing HTLF levels did not assist HIV-1 lacking Vpr [125]. Thus, despite a clear reduction in HLTF levels by HIV-1 Vpr, the impact of HLTF degradation on HIV remained unclear. Subsequent work, however, found HLTF and HIV-1 Vpr to significantly influence viral fitness in primary CD4^+^ T-cell infections. The key to these observations was the use of a Pairwise Growth Competition Assay (PGCA). In brief, traditional comparisons measure the viral output of one virus in a culture versus another virus in a separate culture under similar conditions. PGCA, however, pits the two separate viruses against each other in a single culture to determine which virus becomes the dominant virus detected. PGCA therefore compares viral fitness. An explanation and demonstration of PGCA can be found in [126]. The PGCAs showed a distinct fitness advantage to HIV-1 carrying wild-type Vpr over Vpr null virus or virus carrying Vpr harboring either the Q65R or R80A alterations. These data highlight the importance of HIV-1 Vpr, its ability to interact with DCAF1, and its ability to induce G2 arrest, in HIV-1 infection of primary CD4^+^ T-cells. The PGCAs further revealed that HLTF depletion enhanced both HIV-1 and HIV-1 lacking Vpr but helped the Vpr null virus more. HLTF depletion did not fully restore the infectivity/fitness of HIV-1 lacking Vpr to that of HIV-1, indicating that HLTF is but one of several restriction factors overcome by HIV-1 Vpr [127]. HLTF degradation is not a phenotype shared with HIV-2 Vpr [122].

Exonuclease I (ExoI) is a 5′-3′ exonuclease that contributes to DNA replication, mismatch repair, DSB repair, non-homologous end joining (NHEJ), meiosis, and immunoglobulin maturation (reviewed in [128]). Like what has been observed with HLTF, ExoI is degraded by HIV-1 Vpr, through DCAF1, in a proteasome-dependent manner, and ExoI degradation is not linked to G2 cell cycle arrest. In HEK293T cells ExoI weakly interacts with DCAF1 and DDB1, and HIV-1 Vpr enhances this interaction. However, in vitro ubiquitination assays detect appreciable polyubiquitination of ExoI only in the presence of HIV-1 Vpr, suggesting that ExoI may not necessarily be a native substrate of CRL4-DCAF1. Importantly, spreading infections, initiated with equivalent low multiplicities of infection, revealed ExoI helps to restrict infection of the CEM.SS CD4^+^ T-cell line. Like what was observed with HLTF, ExoI depletion enhanced both HIV-1 and HIV-1 lacking Vpr but helped the Vpr null virus more. Additionally, ExoI depletion did not fully restore the infectivity of HIV-1 lacking Vpr to that of HIV-1, indicating that ExoI is also one of several restriction factors overcome by HIV-1 Vpr [129].

### 3.4. Targets Associated with Myeloid Cell Infection

The primary route of HIV transmission is through sexual intercourse. Myeloid lineage cells, particularly macrophages and dendritic cells, are abundant at mucosal surfaces such as the female reproductive tract. These cells are therefore thought to be among the first cells to encounter HIV (reviewed in [130]). While macrophages and dendritic cells are more resistant to HIV-1 infection, they are nonetheless productively infected and thus contribute to the pathology associated with AIDS in a number of ways (reviewed in [131]), including facilitating transmission to CD4^+^ T-cells (reviewed in [132]). 

In cell culture, HIV-1 Vpr has a modest effect on the infection of cycling primary CD4^+^ T-cells but a much more pronounced effect on HIV infection of primary macrophages and dendritic cells [76,133,134,135,136,137]. HIV-1 Vpr is abundantly packaged in the virion, suggesting an early role during the viral replication cycle. Along these lines, REAF/RPRD2 can restrict HIV-1 reverse transcription in macrophages. Consistent with overcoming this early block, HIV-1 Vpr can initiate REAF/RPRD2 degradation shortly after entry, via DCAF1. REAF/RPRD2 depletion is transient, and REAF/RPRD2 levels rebound within 2 h post entry. The rebound may be due to a cellular response to viral infection as REAF/RPRD2 levels increase in response to the pathogen mimics Poly(I·C) and Lipopolysaccharide (LPS), although not in response to IFN. Basal levels of REAF/RPRD2 are higher in hMDMs and human monocyte derived dendritic cells (hMDDCs) than in cycling primary CD4^+^ T-cells, consistent with a larger role for HIV-1 Vpr in facilitating myeloid lineage cell infection. Interestingly, REAF/RPRD2 is a major component of the Lv2 complex, which restricts specific strains of HIV-1 and HIV-2 harboring certain Env and Gag (core) characteristics. Thus, while the actual block imposed by REAF/RPRD2 is predominately at reverse transcription, the occurrence of the block appears to be dependent on the route of viral entry and/or trafficking of the core. In this manner, REAF/RPRD2 can reduce reverse transcription in otherwise permissive HeLa cells when the cells bear the CD4 receptor to mediate entry of the 89.6 HIV-1 strain lacking Vpr. How variations in Env and Gag (core) structure influence whether or not REAF/RPRD2 negatively impacts reverse transcription is not clear. However, several studies have unveiled a tight interplay between reverse transcription and core/capsid uncoating wherein the two viral replication events influence each other (reviewed in [138]). Thus, REAF/RPRD2 may take advantage of this connection to restrict infection of certain HIV strains. These findings also imply that REAF/RPRD2 may have a physiological role in protecting against specific HIV strains that could be masked in tissue culture experiments utilizing specific lab-adapted or pseudotyped viruses. Of note, though the relative abundance of REAF/RPRD2 is lower in cycling CD4^+^ T-cells, RNAi-mediated depletion of REAF/RPRD2 in the PM1 CD4^+^ T-cell line led to a modest accumulation of cells in G2/M, suggesting that Vpr-facilitated REAF/RPRD2 degradation, regardless of the mechanism of viral entry, may be a contributing factor to the induction of G2 cell cycle arrest [139]. The role of REAF/RPRD2 and Lv2 in retroviral restriction is reviewed in [140].

As discussed in Section 2.2, the endoribonuclease Dicer can be a target of DCAF1-mediated ubiquitination and subsequent degradation, and this may play a role in normal physiological processes. miRNAs are small non-coding RNAs that bind to messenger RNAs (mRNAs) with sufficient complementarity to create a double-stranded RNA molecule that is either degraded or blocked from interacting with cellular ribosomes. Both mechanisms limit translation and thereby participate in post-transcriptional gene silencing. Dicer is a key contributor to miRNA biogenesis. Specifically, Dicer cleaves the pre-miRNA stem-loop structure to generate a miRNA duplex, one strand of which will be used by the RISC complex to identify and silence the target mRNA. Like what has been observed with UNG2, Dicer’s association with CRL4-DCAF1 is enhanced by HIV-1 Vpr, and this leads to increased Dicer turnover. Although HIV-1 Vpr-mediated Dicer depletion was observed in HEK293T cells and the CD4^+^ SupT1 T-cell line, a correlation of Dicer reduction with increased infectivity was only observed in hMDMs. Like with UNG2 depletion, Dicer depletion was linked to HIV-1 Vpr but not its HIV-2 paralogs Vpr or Vpx [141]. Targeting Dicer for degradation would, of course, impact miRNA biogenesis, and the influences of various miRNAs on HIV replication are many (reviewed in [142]).

While Dicer depletion enhanced HIV-1 infection of hMDMs, the enhancement was modest in single-round infections and more pronounced with spreading infections, suggesting that Dicer, and presumably HIV-1 Vpr, have a greater effect on the post-integration steps of viral replication in these cells. Work by Mashiba and Collins et al. identified a macrophage-specific restriction to HIV-1 that blocked the release of virions carrying the native HIV-1 Envelope (Env). This was attributed to increased Env turnover via lysosomal degradation. HIV-1 Vpr was found to overcome this restriction by hindering IFNα production in a manner requiring interaction with DCAF1 [143].

Macrophages and dendritic cells are key antigen-presenting cells (APCs). APCs present microbial peptides to CD4^+^ T-cells via the immunological synapse, an intimate connection of receptor, adhesion, and stimulatory proteins that helps to orchestrate the appropriate adaptive response against a particular pathogen. HIV has evolved to take advantage of this contact-dependent interaction to facilitate cell-to-cell transmission via multiple mechanisms, including the formation of virological synapses (reviewed in [132]). A virological synapse forms when the HIV Env on a budding virion from an infected cell binds to the CD4 receptor on a neighboring uninfected cell. The aforementioned mechanism by which HIV-1 Vpr overcomes Env restriction in macrophages would therefore be expected to contribute to macrophage-mediated HIV transmission to CD4^+^ T-cells via the virological synapse. Indeed, follow-up work demonstrated this to be the case in co-cultured primary immune cells [144].

Several proteins have been identified that restrict HIV-1 Env production in macrophages and are countered by HIV-1 Vpr. IFITM3, an IFN-inducible transmembrane protein capable of shuttling viruses to lysosomes for destruction, was implicated in Env restriction in macrophages, and HIV-1 Vpr overcame *ifitm3* gene expression through degradation of the transcriptional regulator TET2 [145]. However, other work found IFITM3 levels in hMDMs exhibited donor variability and reduction in IFITM3 was not consistently observed in the presence of HIV-1 Vpr, suggesting that IFITM3 may not be a universally employed HIV-1 Env restriction in macrophages. The macrophage-specific HIV-1 Env restriction was instead linked to the mannose receptor (MR). Through synergistic, but separate, mechanisms HIV-1 Vpr and HIV-1 Nef coordinate a maximal reduction in MR to alleviate lysosomal degradation of HIV-1 Env in hMDMs. HIV-1 Vpr specifically contributed to HIV-1 Env protection by transcriptionally repressing MR gene (*mrc1*) expression, and this was associated with a requirement of HIV-1 Vpr to engage DCAF1 [146]. 

Recent work identified PU.1 as a target of HIV-1 Vpr. Importantly, PU.1 degradation potentially unifies the macrophage-specific HIV-1 Env antagonization by IFN, IFITM3, and MR. PU.1 is the master myeloid cell transcription factor and is critical for both macrophage differentiation and function [147,148]. As such, PU.1 plays vital roles in both the IFN response and IFN production in macrophages. Thus, HIV-1 Vpr-mediated loss of PU.1 would contribute to reduced IFNα production. Further, single-cell RNA sequencing revealed that HIV-1 Vpr reduced the expression of PU.1-responsive genes, including *ifitm3* and *mrc1*, in hMDMs. Like what was observed with the IFN, IFITM3, and MR restrictions, association with DCAF1 was required for PU.1 degradation by HIV-1 Vpr. Interestingly, PU.1 did not associate well with either HIV-1 Vpr or DCAF1 individually but did so when both Vpr and DCAF1 were similarly abundant. This may be due to PU.1’s association with TET2 [149]. As mentioned earlier, TET2 is a native interaction partner for DCAF1 [53] and is targeted by HIV-1 Vpr to repress *ifitm3* gene expression [145]. Therefore, mutual interaction of PU.1, DCAF1, and Vpr with TET2 may serve as a means for Vpr to gain access to PU.1. Other consequences of PU.1 and TET2 loss to cellular gene expression are discussed in Section 3.5.

LAPTM5 was also identified as a macrophage-specific restriction factor that antagonizes HIV-1 Env. LAPTM5 redirects HIV-1 Env from the secretory pathway to the lysosomal compartment for destruction in hMDMs. HIV-1 Vpr counters LAPTM5 via DCAF1 in a manner requiring a functional proteasome [150]. Both MR and LAPTM5 are abundant in macrophages relative to CD4^+^ T-cells and various permissive cell lines, further implicating their role in a macrophage-specific restriction. Of note, HIV-1 Vpr was also found to inhibit LAPTM5 restriction of HIV-1 Env in hMDDCs [151], although that work was not as extensive as that characterizing the restriction in hMDMs. Whether the PU.1/MR and LAPTM5 restrictions overlap or intersect has not yet been clarified. It would be interesting to see the role of HIV Nef, if any, on the LAPTM5 restriction given its contribution to disabling the MR restriction. It would also be interesting to understand how LAPTM5 responds to IFNα which, as described above for the earlier work, overcame HIV-1 Vpr-mediated rescue of the HIV-1 Env restriction in hMDMs [143].

The canonical function of dendritic cells is to act as sentinel cells that survey various tissues and alert the adaptive response to a microbial threat. These roles make dendritic cells excellent for viral dissemination within the host. Dendritic cells, relative to macrophages and activated CD4^+^ T-cells, are the most resistant to HIV-1 infection but are productively infected. The activity of intrinsic antiviral defenses, such as SAMHD1 (discussed in Section 4.2), play a role in resisting HIV-1 infection, but a slew of other mechanisms remains incompletely understood. Like what has been described above for macrophage infection, a lysosome-dependent restriction to HIV-1 Env was identified in hMDDCs and was also countered by HIV-1 Vpr. Unlike the macrophage work, however, interaction of HIV-1 Vpr with DCAF1 was not necessary for countering the block [152]. Other work found HIV-1 Vpr to enhance hMDDC infection in a DCAF1-dependent manner by increasing HIV-1 LTR-driven gene expression rather than influencing HIV Env processing [153]. Of note, this phenotype is reminiscent of that observed after CCDC137 depletion (discussed in Section 3.2). It is also possible that the aforementioned HIV-1 Vpr-mediated degradation of PU.1 contributes to enhanced HIV-1 LTR-driven gene expression [154], but such a role has not yet been fully established [149]. Though the precise mechanism by which HIV-1 Vpr enhanced gene expression in hMDDCs is not known, several targets have been identified, the loss of which may contribute to such a phenotype. These will be discussed next. 

### 3.5. Targets Associated with HIV or Host Cell Gene Expression

Retroviruses, such as HIV, reverse transcribe their RNA genome into a dsDNA copy. This viral DNA is subsequently integrated into the host cell genome (now termed a provirus). The provirus serves as the template for the synthesis of viral mRNAs as well as new copies of the viral genome. Establishment of a provirus is critical to the HIV-replication cycle, and proviruses contribute to HIV persistence within the host.

Silencing of HIV proviruses can have both detrimental and beneficial outcomes for viral replication. From the perspective of the host, halting HIV gene expression limits viral spread and thereby limits overall pathology. From the perspective of the virus, however, silencing in the context of latency can enable viral persistence and re-emergence under favorable conditions. 

Chromatin remodeling complexes introduce post-translational modifications to histone proteins to either encourage or discourage DNA compaction. Histone Deacetylases (HDACs) are a class of proteins that remove acetyl groups from histone protein tails and contribute to the formation of heterochromatin, regions of DNA that are tightly compacted. Thus, HDACs reduce gene expression by reducing the accessibility of certain genes to associated transcription factors. In the context of HIV infection, viral dismantling of HDAC complexes favors efficient gene expression. One phenotype ascribed to HIV-1 Vpr is its ability to enhance HIV gene expression. Work by several labs has identified multiple CRL4-DCAF1-dependent targets of HIV-1 Vpr, the loss of which would theoretically foster a cellular environment conducive to HIV gene expression.

Zip and its isoform sZip are degraded by HIV-1 Vpr in a DCAF1-dependent manner in HeLa cells. Zip and sZip degradation is independent of G2 cell cycle arrest. Zip, a component of the NuRD deacetylase complex, contributes to transcriptional repression through DNA compaction. Loss of Zip would therefore be assumed to be favorable for HIV gene expression. However, HIV-1 Vpr-mediated degradation of Zip did not influence LTR-driven transcription in HeLa cells [155]. Further, sZip, which may act in opposition to Zip [156], is also degraded by HIV-1 Vpr, a finding that is counterintuitive as preservation of sZip would be assumed to benefit HIV gene expression. Likewise, SIRT7, a member of the sirtuin protein family with histone deacetylase activity, is degraded by HIV-1 Vpr in a DCAF1-dependent manner. Reduction of SIRT7 by HIV-1 Vpr was observed in HEK293T cells, one of many immortalized cell lines wherein HIV-1 Vpr is dispensable for viral replication. SIRT7 depletion was also unlinked from G2 cell cycle arrest [157]. It is possible that an impact of Zip/sZip and SIRT7 on HIV gene expression may be more apparent in contexts where HIV-1 Vpr enhances infection, such as spreading infection of myeloid lineage cells or carefully characterized and titrated infections in cycling CD4^+^ T-cells [76]. It is also possible that Zip/sZip and SIRT7 may be collateral damage from HIV-1 Vpr-mediated degradation of other chromatin-remodeling proteins like those discussed below.

HIV-1 Vpr can be recruited to chromatin via DCAF1. As described in Section 2.2, DCAF1 can directly interact with the tail of histone H3. HIV-1 Vpr, in complex with DCAF1, targets class I HDACs, including those present on the HIV-1 LTR, for degradation. HIV-1 Vpr-dependent reduction of class I HDACs was observed in HeLa cells and was independent of G2 cell cycle arrest. Importantly, reduction in chromatin-bound HDACs, particularly HDAC3, by HIV-1 Vpr led to latent viral genome reactivation in hMDMs [158], in the J-Lat 10.6 cell line model of latency, and in Peripheral Blood Mononuclear Cells (PBMCs) from HAART-treated HIV-1-infected individuals [159].

CTIP2 recruits chromatin-remodeling proteins to induce heterochromatin formation and, thus, gene silencing. CTIP2 has been shown to recruit the aforementioned NuRD complex [160], of which the type I HDACs HDAC1 and HDAC2 are components, to suppress HIV-1 gene transcription [161,162]. CTIP2 is degraded by HIV-1 Vpr in both the CD4^+^ Jurkat T-cell line and in primary CD4^+^ T-cells, and HIV-1 Vpr interaction with DCAF1 is required for the degradation. Further, HIV-1 Vpr-mediated degradation of CTIP2 promoted HIV gene expression reactivation in a microglial model of latency [163]. HIV latency in CD4^+^ T-cells and microglia, a macrophage-like cell of the central nervous system, presents a major hurdle to viral clearance.

While the elimination of the targets described above may contribute to increased expression from integrated proviruses, not all viral DNA successfully integrates. Indeed, several unintegrated viral DNA species are transiently present in primary immune cells. These short-lived extrachromosomal viral DNAs can persist for a few hours to a few weeks depending on the cell type (cycling CD4^+^ T-cells < resting CD4^+^ T-cells < macrophages) and the nature of the extrachromosomal viral DNA (e.g., linear unintegrated DNA < 1- or 2-LTR circles) (reviewed in [91]). Interestingly, the expression from these unintegrated viral DNAs is considerably less than integrated proviruses despite often carrying the same information, and this is attributed to rapid chromatinization accompanied by histone-silencing modifications [164,165]. Work by Dupont et al. found that HIV-1 Vpr initiates degradation of SLF2 via CRL4-DCAF1. Degradation of SLF2 prevents the recruitment of Smc5/6, which in turn act to compact and therefore silence gene expression from unintegrated viral DNA. HIV-1 Vpr-mediated antagonism of SLF2/Smc5/6 is independent of G2 cell cycle arrest. Like G2 cell cycle arrest, however, SLF2 degradation is a phenotype also shared by HIV-2 Vpr, indicative of an evolutionarily conserved function. Vpr-mediated SLF depletion and increased unintegrated viral DNA gene expression was observed in CD4^+^ T-cell lines and in primary CD4^+^ T-cells [166]. In all, these findings suggest an important role for unintegrated viral DNA in HIV biology that has yet to be fully elucidated. 

In addition to enhancing HIV gene expression, HIV-1 Vpr can modulate the expression of host cell genes to benefit viral replication. HIV-1 Vpr, through interaction with CRL4-DCAF1, degrades the DNA-modifying dioxygenases TET1–TET3. TET2 is the most abundant of the three in the THP-1 monocytic cell line and, as alluded to in Section 2.2, binds to DCAF1 as part of normal physiological responses. This association leads to monoubiquitination of TET2 by CRL4. Monoubiquitnation increases TET2 association with chromatin [167], enabling TET2 to recruit members of the type I HDACs to silence the IL-6 promoter. HIV-1 Vpr, and HIV-2 Vpr, re-orient TET2 to the C-terminal region of DCAF1 to favor poly-, rather than mono-, ubiquitination, thereby facilitating TET2 degradation via the proteasome. HIV-1 Vpr reduced TET2 levels in HEK293T cells and PBMCs independent of G2 cell cycle arrest. HIV-1 Vpr also decreased TET2 levels in monocytes of the THP-1 cell line as well as in hMDMs. Importantly, TET2 depletion was linked to increased IL-6 production and enhanced viral spread of HIV-1 in a Vpr-dependent manner in hMDMs [168]. Macrophages are a major source of IL-6 production during human HIV infection. IL-6 contributes to the persistent inflammation that exacerbates AIDS and non-AIDS diseases (e.g., increases in all-cause mortality observed in HIV-infected individuals receiving treatment (reviewed in [169]). Thus, through TET2 degradation, HIV-1 Vpr can upregulate IL-6 while simultaneously downregulating IFITM3 to maximize macrophage infection.

As discussed in Section 3.4, HIV-1 Vpr can degrade the master myeloid cell transcription factor PU.1 to hinder IFN responses and reduce *mrc1* and *ifitm3* expression. Interestingly, single-cell RNA sequencing of HIV-1 exposed hMDMs revealed a reduction of PU.1 responsive gene transcripts in hMDMs wherein *tat* and *gag* were not expressed. Because Tat and Gag are late viral gene products, their detection is indicative of gene expression from a successfully integrated provirus [149]. Thus, the downregulation of PU.1 responsive gene transcripts in genuinely infected cells triggers a similar repression in uninfected bystander cells. This may be due to a failure of genuinely infected cells to produce cytokines that would activate PU.1-mediated responses in uninfected bystander cells. Alternatively, PU.1 may be degraded by Vpr introduced as a result of a failed infection or the uptake of soluble extracellular Vpr released from infected cells. Regardless of the mechanism by which PU.1 responses are suppressed in bystander cells, these findings add to an expanding role for HIV-1 Vpr-mediated PU.1 degradation in promoting HIV replication. Of note, PU.1 depletion is a phenotype shared with HIV-2 Vpr [149]. While HIV-2 Vpr is dispensable for macrophage infection, this conserved function further underscores the importance of PU.1 antagonism to HIV replication. 

HIV-1 Vpr can also act upstream of chromatin remodeling to influence host gene transcription by blocking the nuclear import of proteins that act to establish an antiviral state. HIV-1 Vpr interacts with KPNA1 (importin α) to inhibit activation of the innate immune response by the transcription factors IRF3 and NFκB. Nuclear translocation of IRF3 and NFκB can be induced by several Pathogen Associated Molecular Patterns (PAMPs) (e.g., viral RNA, cytosolic DNA, and LPS). HIV-1 Vpr, via its interaction with KPNA1, broadly blocks nuclear translocation of IRF3 and NFκB regardless of the inductive PAMP used, including the viral genome itself. HIV-1 Vpr’s association with DCAF1 is required for the effect via an unknown mechanism that does not rely on degradation of KPNA1. The block is observed in both hMDMs and the THP-1 monocyte-macrophage cell line. Interestingly, this did not appear to occur in primary CD4^+^ T-cells where HIV-1 Vpr was ineffective at rescuing HIV-1 from responses elicited by cGMP, a signaling molecule produced in response to cytosolic DNA, suggestive of uninhibited IRF3 localization to the nucleus [170]. These observations are reminiscent of several earlier works linking HIV-1 Vpr’s accumulation at the nuclear envelope with enhanced spreading infection in macrophages [137,171]. Thus, this work may explain earlier observations that were interpreted as HIV-1 Vpr enhancing nuclear import of viral DNA. Of note, other work found IRF3 to be degraded by HIV-1 Vpr and Vif via the proteasome in the dividing cell lines: HeLa, HEK293, and Jurkat [172]. HIV may therefore employ different mechanisms to antagonize IRF3 function in a context-dependent manner. In this fashion, HIV-1 Vpr has been shown to both inhibit and activate NF-κB, the latter being favorable for enhanced HIV LTR transcription. Thus, HIV’s manipulation of NF-κB may be context- and timing-dependent, a concept possibly reflected in work by Hotter et al. demonstrating assay-dependent activation or inhibition of NF-κB by diverse SIV Vprs [173].

### 3.6. Other Targets

Like IL-6, Tumor Necrosis Factor (TNF) is a pro-inflammatory cytokine that is elevated in HIV-1 infection and contributes to pathology (reviewed in [174]). Work by Roesch et al. found that de novo produced, rather than virion incorporated, HIV-1 Vpr contributed to an increase in TNF levels in the MT4 CD4^+^ T-cell line and in primary CD4^+^ T-cells. RNAi-mediated depletion of DDB1 and TAK1 counteracted HIV-1 Vpr’s ability to enhance TNF production. TAK1 is a kinase whose activation indirectly activates NF-kB. HIV-1 Vpr had previously been found to bind TAK1 [175], supporting the notion that HIV-1 Vpr complexes with DDB1 and TAK1 to enhance TNF production. Further supporting this, HIV-1 Vpr Q65R, which is defective in DCAF1 binding, yielded reduced, albeit not completely abrogated, TNF production. Moreover, the ability of HIV-1 Vpr to enhance TNF production correlated with Vpr’s ability to induce G2 cell cycle arrest, although the temporal order of these events was not determined. In all, these data support HIV-1 Vpr interaction with CRL4 as contributing to TNF production through a mechanism that remains to be fully delineated. Of note, RNAi-mediated depletion of DDB1 and TAK1 impaired TNF release after infection with Vpr null virus, suggesting that infection-stimulated TNF release may be another physiological function of DDB1 that is enhanced by HIV-1 Vpr [176].

As discussed in Section 1.4, the cytidine deaminase APOBEC3G potently inhibits HIV replication and is countered by HIV Vif. HIV-1 Vpr was shown to reduce APOBEC3G levels, independent of Vif, via DCAF1 and proteasomal degradation in HEK293T cells and in the H9 CD4^+^ T-cell line. Importantly, HIV-1 Vpr-mediated degradation resulted in reduced APOBEC3G incorporation into virions in the absence of Vif, though not as robustly as with Vif [177]. While the redundancy of APOBEC3G elimination through HIV-1 Vpr is interesting, it is not surprising given that APOBEC3G is the dominant restriction to HIV infection in primary CD4^+^ T-cells and has been under positive selection for 33 million years (reviewed in [178]). Similarly, HIV-1 Vif demonstrates some functional overlap with HIV-1 Vpr by inducing G2/M cell cycle arrest (reviewed in [179]) and antagonizing DNA repair proteins [180], albeit through mechanisms distinct from those employed by HIV-1 Vpr. While it is not yet known whether these functions are synergistic or compensatory, their redundancy further implicates an in vivo significance of these phenotypes to HIV infection. 

Another phenotype attributed to HIV-1 Vpr is the ability of the viral protein to cause cell death, particularly apoptotic cell death (reviewed in [181]). Interestingly, this is not a phenotype shared with HIV-2 Vpr or Vpx [182]. The ability of HIV-1 Vpr to cause cell death is, in part, credited to its ability to disrupt the mitochondrial outer membrane (MOM) integrity, leading to the release of proapoptotic factors such as cytochrome *c* [183]. Mfn2 is a protein involved in trafficking of vesicles from the mitochondria-associated-membrane of the endoplasmic reticulum (ER) to the MOM. HIV-1 Vpr was found to associate with this region of the ER possibly through a C-terminal hydrophobic region, residues 55–83. Further, HIV-1 Vpr was found in both transport vesicles destined for the MOM and in the MOM. HIV-1 Vpr promoted the degradation of Mfn2 in a CRL4-DCAF1-dependent manner. Importantly, Mfn2 depletion led to mitochondrial membrane permeability and cell death in the HEK293 and CD4^+^ SupT1 T-cell lines [184]. Of note, residues 55–83 of HIV-1 Vpr have also been associated with neuropathology attributed to extracellular/soluble Vpr entering various cell types and exerting detrimental effects such as the induction of apoptosis (reviewed in [185]).

APC1 is a component of the Anaphase Promoting Complex/Cyclosome (APC/C), an E3 ubiquitin ligase that controls entry into S phase of the cell cycle and regulates progression through the M phase. APC1 is degraded by HIV-1 Vpr in a DCAF1-dependent manner. APC1 degradation by HIV-1 Vpr was not linked to G2 cell cycle arrest, and it did not impact HIV infection of either primary CD4^+^ T-cells or hMDMs. While the benefit of APC1 degradation to HIV replication is not clear, some evidence points to a potentially important contribution. APC/C is a target manipulated by different viruses (reviewed in [186]). Additionally, Vpr from several primary HIV-1 isolates degraded APC1. This suggests reduction of APC1 is a conserved function of HIV-1 Vpr, though APC1 degradation was not observed with Vpr from the prototypic NL4-3 HIV-1 lab strain [187]. 

HIV-1 Vpr engages the EDVP HECT domain E3 ubiquitin ligase complex, through DCAF1, to enhance turnover of TERT in HeLa cells and impair telomerase activity in the CD4^+^ T-cell lines Jurkat and SupT1 in a manner independent of G2 cell cycle arrest [188]. TERT is the catalytic subunit of telomerase, which acts to maintain chromosome length that would otherwise be lost over subsequent cellular divisions due to limitations imposed on DNA polymerase during lagging strand synthesis. HIV-1 Vpr-mediated loss of telomerase function may be a contributing factor to the higher risk of aging-associated complications observed in HIV-infected individuals [189]. How loss of TERT may impact HIV replication is not clear.

HIV-1 Vpr also engages the EDVP HECT domain E3 ubiquitin ligase complex through DCAF1 to enhance turnover of CP110. Loss of CP110 in HEK293, HeLa, and CD4^+^ MT4 T-cell lines leads to centriole elongation, centrosome amplification, and increased nucleation of cytoplasmic microtubules. Though perturbation of centrosome homeostasis through this mechanism was also not linked to G2 cell cycle arrest [190], HIV-1 Vpr-mediated stabilization of microtubules via this mechanism may contribute to a number of viral replication events (e.g., delivery of the viral core to the nucleus) that rely on stable microtubules (reviewed in [191]).

HIV-1 Vpr’s modulation of the cytoskeleton may contribute to the increased risk of cancer in HIV-infected individuals. In the context of human infection, 1–5% of CD4^+^ T-cells exhibit overduplication of centrosomes possibly contributing to aneuploidy. HIV-1 Vpr was found to engage PLK4, a kinase involved in centriole duplication. HIV-1 Vpr enhanced PLK4 activity, and this required interaction with DCAF1 and DDB1 but not Cul4A or Cul4B. PLK4 was not ubiquitinated, and the aforementioned turnover of CP110 does not appear to be involved. This effect was independent of G2 cell cycle arrest. Whether other components of the EDVP complex (e.g., DYRK2) are involved was not tested [192]. How increased PLK4 activity impacts HIV replication is unclear, but increased activity of PLK4 may also influence microtubule-based HIV functions.

**Figure 3:**
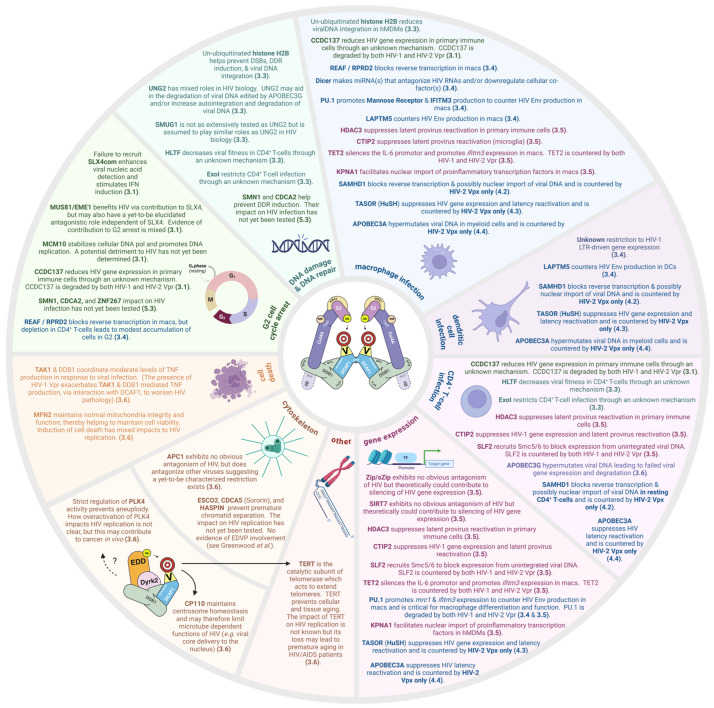
Summary of DCAF1-dependent HIV Vpr and Vpx targets. Cellular targets are grouped by the cellular and/or viral function/phenotype for which they are associated. Each target is accompanied by a brief summary of their possible role in antagonizing HIV replication. Of note, targets may be associated with more than one function/phenotype, and color coding is based on the function/phenotype for which they are initially described. For each category, targets are generally listed in the order in which they appear in the review. Targets of HIV Vpr are discussed in Section 3 and Section 5. Targets associated with HIV-2 Vpx are discussed in Section 4. (**#**.**#**) denotes the corresponding subsection in which that target is discussed. DDR = DNA Damage Response. This figure was created with BioRender.com.

DCAF1-dependent targets of HIV Vpr and Vpx are summarized in Figure 3.

## 4. CRL4-DCAF1 and HIV-2 Viral Protein X

The most well-recognized phenotype of HIV-2 Vpx is its ability to markedly enhance infection of myeloid lineage macrophages and dendritic cells [193] through interactions with CRL4-DCAF1 [194,195,196]. Vpx is indispensable for HIV-2 replication in these cells, and Vpx greatly enhances HIV-1 infection of myeloid cells despite HIV-1 having Vpr [195]. These earlier findings suggested that HIV-2 Vpx either more efficiently performs the same function as HIV-1 Vpr or performs a separate function entirely. In the following sections, we discuss the interaction of HIV-2 Vpx with the CRL4 complex and the targets of this interaction identified to date that reveal how Vpx enables these favorable phenotypes to HIV replication.

### 4.1. Vpx, CRL4-DCAF1, and Target Protein Interactions

HIV-2 Vpx is a 112-amino-acid, 18-kDa protein. Owing to Vpx likely having evolved from a common ancestor of Vpr, Vpx structure strongly resembles that of Vpr. Vpx forms an unstructured N-terminal tail, a three-helix bundle, and an unstructured C-terminal tail. Considering variation in the available computational and structural data, the three α helices of Vpx approximately map to amino acid positions 18–37, 42–56, and 64–85, respectively. However, unlike Vpr, Vpx lacks a hydrophobic cleft due to the presence of bulky side chains, including Vpx residues W49 and Y71. Vpx also forms a longer insert loop than Vpr [60]. 

As with Vpr, the highly conserved Wx4Φx2Φx3AΦxH motif is essential for proper Vpx function. In Vpx, this motif maps to α-helix 1 (residues 24–39) and is required for Vpx-DCAF1 interactions. The Vpx W24A alteration interacts with DCAF1 and SAMHD1 but failed to effectively degrade SAMHD1. SAMHD1 is a target of Vpx discussed in Section 4.2. This suggests that the W24 residue plays an important, but not yet completely characterized, role in this function. The importance of the conserved motif is additionally demonstrated through Vpx alterations V29S, I32S, H39A, and the A36S/V37S double alteration, which all demonstrated attenuated interaction with DCAF1 to some degree (50–90%). As expected, these four alterations exhibited impaired ability to degrade SAMHD1. Of note, all of these alterations maintained the ability to localize to the nucleus, a phenotype common between Vpr and Vpx [61].

Similar to HIV-1 Vpr, HIV-2 Vpx contains a highly conserved HHCH/HHCC zinc-binding motif that maps to amino acid positions 39, 82, 87, and 89. SIVmac239 Vpx (a close ortholog to HIV-2 Vpx) alterations H39A, H82A, C87S, and C89S were defective in inducing SAMHD1 degradation. These alterations exhibited an 80–95% decrease in binding DCAF1 but retained the ability to bind SAMHD1 [62]. Thus, as with the HHCH domain of HIV-1 Vpr, the HHCC domain of Vpx proteins favors a zinc-dependent mechanism for Vpx assembly with CRL4-DCAF1. Of note, the single Vpx H82A alteration is sufficient to hinder Vpx-mediated antagonism of APOBEC3A [197], a less extensively studied target of Vpx-mediated degradation (described in Section 4.4).

Like Vpr, α-helix 3 of Vpx is important for Vpx-DCAF1 interactions ([60] and reviewed in [198]). Consistent with this statement, Vpx alteration Q76R, mapping to a highly conserved residue in α-helix 3 of Vpx, was defective in binding with DCAF1 [194], resulting in Vpx failing to promote SAMHD1 degradation [199]. 

Schwefel et al. found that SIVsmm Vpx (a close ortholog to HIV-2 Vpx), contains the amino acid sequence PPGNSGEET which maps to residues 9–17 of the N-terminal tail. This sequence is conserved in HIV-2 Vpx and contains residues E15 and E16, which form salt bridges with residues R607 and R617 of SAMHD1. Vpx residues W24, Y66, and Y69 mediate bridging interactions by forming hydrophobic interactions with R617, K622, and V618 of SAMHD1, respectively, and hydrogen bonds with N1135, D1092, and E1091 of DCAF1, respectively. K622 of SAMHD1 and D1092 of DCAF1 also hydrogen bond with each other, forming the only direct interaction between SAMHD1 and DCAF1 [200]. In contrast to Vpr, the importance of the C-terminal tail is less well-understood, but alterations in the poly-proline motif (amino acid positions 103–109) may enable efficient Vpx translation [201], contribute to Vpx oligomerization, and influence SAMHD1 degradation [202].

**Figure 4:**
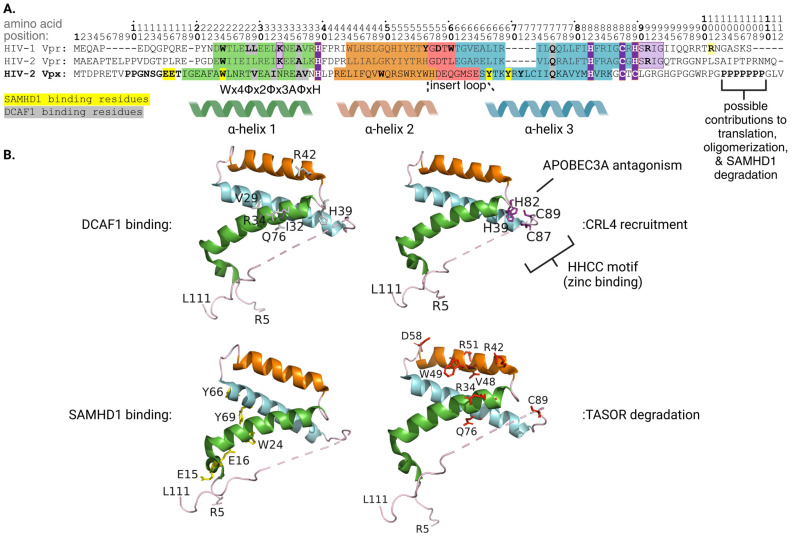
Interaction properties of HIV-2 Vpx. (**A**) Amino acid sequences of HIV-1 Vpr from HIV-1 89.6 (accension Q73369) and HIV-2 Vpr and Vpx from HIV-2 ROD (accension P06938 and P06939, respectively). (**B**) Ribbon and stick representations of SIVsmm Vpx (a close ortholog of HIV-2 Vpx) (from PDB 4CC9). Key residues, motifs, and domains for the indicated interactions are color coded and/or bolded and/or labeled for each panel. Ribbon and stick representations were created with the PyMOL Molecular Graphics System, Version 2.5.5. This figure was assembled with BioRender.com.

TASOR (discussed in Section 4.3), like SAMHD1, is targeted by Vpx through CRL4-DCAF1. However, the interactions necessary for degradation of the two targets differ. While Vpx W49A, C89A, and D58A alterations had impaired abilities to degrade both SAMHD1 and TASOR, other alterations, including R34A, R42A, V48A, and R51A, showed impaired TASOR degradation but remained capable of triggering SAMHD1 degradation. Vpx R34A, R42A, and R51A were also less effective at binding DCAF1, and this was exacerbated with the Vpx R34A/R42A double alteration. However, all four alterations retained the ability to interact with TASOR. Overall, these findings indicate that the residues required for TASOR and SAMHD1 degradation do not necessarily overlap and that a strong binding affinity between Vpx and DCAF1 is essential to induce TASOR degradation, relative to that required for SAMHD1 degradation [203]. The interaction properties of HIV-2 Vpx are presented in Figure 4.

### 4.2. Vpx and SAMHD1

HIV-2 Vpx’s ability to enhance infection of myeloid lineage cells was linked to overcoming a block to reverse transcription, although earlier work found Vpx overcame a block that acted predominantly to restrict nuclear import of viral DNA (reviewed in [17] and [18]). SAM and HD domain-containing protein 1 (SAMHD1) was identified as the major restriction factor limiting HIV replication in myeloid lineage cells that is countered by Vpx [199,204]. SAMHD1 is either absent or inactivated, via phosphorylation at Threonine 592 (T592), in cells permissive to HIV infection [205,206]. Vpx engages CRL4-DCAF1 to induce proteasomal degradation of SAMHD1, and SAMHD1 does not appear to be a native substrate of CRL4-DCAF1 [207]. Interestingly, HIV-1 Vpr and HIV-2 Vpr do not have the capacity to efficiently degrade SAMHD1, but several related SIV Vpx and Vprs do [208]. Subsequent work found SAMHD1 also restricts HIV-1 infection in resting CD4^+^ T-cells [209]. Through its phosphohydrolase activity, SAMHD1 depletes the free dNTP pool in non-dividing cells, thereby limiting viral DNA synthesis and thus the observed block to reverse transcription [210]. HIV-1 may partially compensate for the reduced availability of dNTPs via its RT having greater affinity for dNTPs and faster enzymatic activity [211]. Unlike terminally differentiated macrophages and dendritic cells or resting CD4^+^ T-cells, actively dividing CD4^+^ T-cells would need a sufficient supply of dNTPs to carry out DNA replication. SAMHD1 is therefore inactivated in these cells. SAMHD1 phosphohydrolase activity can be inactivated by T592 phosphorylation [212] and/or oxidizing conditions that promote a monomeric (as opposed to tetrameric) state of SAMHD1 [213] in a cellular context-dependent manner. Interestingly, a considerable body of work hinted at an additional restriction imposed by SAMHD1 that is dNTP-independent [214,215], is controlled by T592 phosphorylation and/or the cellular redox state, and acts to restrict nuclear import of viral DNA [216,217,218,219]. Indeed, this additional restriction was recently confirmed by Guo et al. [220]. Vpx does not appear to discriminate between the unphosphorylated and phosphorylated forms of SAMHD1 [205]. Of note, SAMHD1 has several physiological functions. Thus, its loss may influence HIV-2 pathology beyond facilitating viral replication (reviewed in [221]). 

### 4.3. Vpx and the HuSH Complex

The identification of SAMHD1 as a major target for HIV-2 Vpx-mediated degradation helped to explain the previously elusive mechanism by which Vpx markedly enhanced myeloid lineage cell infection. However, SAMHD1 depletion by Vpx could not fully explain all the phenotypes associated with Vpx. For example, despite being dispensable for replication in many non-myeloid cell lines, Vpx is required for efficiently spreading infection in peripheral blood lymphocytes [222] wherein SAMHD1 is primarily in the inactive state. Furthermore, any of a select set of point mutations confer SIVmac239 Vpx (a close ortholog to HIV-2 Vpx) the ability to overcome an additional restriction to HIV-1 reverse transcription in resting CD4^+^ T-cells that is independent of that induced by SAMHD1 [223]. Additionally, Vpx rescues HIV-1 infection of hMDDCs from an IFNβ/LPS inducible restriction that cannot be overcome by increasing the dNTP pool and does not require DCAF1 [214,224] and reviewed in [19].

RNAi-based loss-of-function assays identified TASOR (FAM208A), a component of the Human Silencing Hub (HuSH) complex, as an interaction partner of Vpx. Vpx reduced TASOR levels in a DCAF1 and proteasome-dependent manner, and TASOR depletion by Vpx was observed in both primary CD4^+^ T-cells and hMDMs. As its name implies, the HuSH complex promotes gene silencing by recruiting methyltransferases. Methyltransferases transfer methyl groups to cytosine residues in DNA to favor gene silencing. HuSH had previously been shown to suppress proviruses, retrotransposons, and LINE-1 elements (reviewed in [225]), suggesting Vpx antagonization of HuSH could enhance HIV gene expression. Indeed, Vpx-mediated TASOR depletion enhanced both HIV-1 and HIV-2-*related* SIV LTR-driven gene expression in the CD4^+^ Jurkat T-cell line. Further, Vpx-mediated depletion of TASOR encouraged reactivation of HIV-1 gene expression in the J-LAT cell line model of latency. This effect was independent of SAMHD1 degradation and is consistent with the observations having been made in Jurkat-based cell lines that do not produce SAMHD1. While HuSH component depletion by Vpx was not a phenotype shared with HIV-1 Vpr, it is shared with several related SIV Vpx and Vpr proteins [226,227]. It is likely that HIV-1 relies on other mechanisms to enhance gene expression, such as those discussed in Section 3.5. Interestingly, Vpx may take advantage of a normal physiological interaction of DCAF1 with TASOR, though TASOR does not appear to be a native substrate of CRL4-DCAF1, at least in regard to proteasomal degradation [203]. 

### 4.4. Vpx and APBOBEC3A

APOBEC3A is another member of the cytidine deaminase family that is abundant in monocytes, the blood precursor cells to tissue macrophages and dendritic cells, and to a lesser extent in macrophages and dendritic cells. APOBEC3A restricts spreading HIV-1 infection in myeloid lineage cells, and this correlates with reduced infectious viral DNA [228,229], consistent with the cytidine deaminase activity of APOBEC family members. Vpx promotes APOBEC3A degradation in a proteasome-dependent manner [197,229], presumably through CRL4-DCAF1, though this has not been directly tested. Relative to myeloid lineage cells, APOBEC3A levels are lower in primary naïve and activated CD4^+^ T-cells. Nonetheless, APOBEC3A has also been shown to bind to the NF-κB/Sp1 binding region of the HIV-1 LTR to recruit the cellular KAP1 and HP1 chromatin remodeling proteins, thereby suppressing HIV-1 proviral gene expression in both J-LAT and experimentally induced primary CD4^+^ T-cell models of latency [230]. Presumably, Vpx would act to counter this restriction as well and counter both restrictions in the context of infection with its native virus HIV-2. It is interesting that Vpx’s antagonism of APOBEC3A to both enhance myeloid cell infection and possibly HIV gene expression is mirrored in its antagonism of SAMHD1 and HuSH, respectively. DCAF1-dependent targets of HIV Vpr and Vpx are summarized in Figure 3.

## 5. Discussion—Themes and Questions

The findings discussed in this review have led to the emergence of several themes and questions. These are summarized in the proceeding subsections.

### 5.1. The CRL4-DCAF1 Complex Is Complex

Conventional representations of CRL4, especially in the context of HIV Vpr/x, are useful for obtaining a fundamental understanding of function, but are likely insufficient for elaborating nuances that could play key physiological and pathological roles. Cul4A and Cul4B are not entirely functionally redundant. While the two Cul4 types are interchangeable for Vpr and Vpx function in cell culture, is there an in vivo cellular context wherein Vpr/x relies more heavily on one Cul4 type over the other? In addition to harboring an extra NLS, the N-terminus of Cul4B may contribute to unique substrate recognition [231]. Are these differences exploited by Vpr/x? Given findings that support CRL4-DCAF1 being maximally functional in a dimeric state, do CRL4A-DCAF1-DCAF1-CRL4B heterodimers form in vivo? Would Vpr/x have “the best of both worlds” by accessing such a heterodimeric complex? HIV-2 harbors both Vpr and Vpx. Do these viral proteins compete for access to CRL4? Do they each bind entirely separate complexes or function “side-by-side,” with each choosing one monomer of a CRL4 dimer? Of note, some HIV-2 isolate Vprs are less stable than HIV-1 Vpr or HIV-2 Vpx, and this has been attributed to an incomplete HHCH/HHCC motif important for CRL4 interaction and viral protein stability [232]. Might HIV-2 have evolved an optimal ratio of Vpr to Vpx for CRL4-DCAF1 engagement? 

The capacity of CRL4-DCAF1 to form multimeric complexes imparts unique regulation that may not necessarily be shared by other CRLs. Specifically, CRL4-DCAF1 can assemble in an inactive tetrameric state. While neddylation plays a role in modulating this multimerization, HIV-1 Vpr binding to UNG2 favors the active dimeric form of CRL4 to possibly liberate the complex from the restrictive tetrameric state. Importantly, this activation may not necessarily require neddylation [40]. It has been speculated that this mechanism may explain observed spontaneous CRL4 activity in the absence of neddylation [233,234,235]. While neddylation has been shown to be required for Vpr/x function through CRL4, is there a context wherein viral protein binding of target substrates is sufficient to induce ubiquitination independent of the requirement for neddylation? Of note, Nekorchuk et al. found inhibition of neddylation rescued endogenous UNG2 from HIV-1 Vpr-mediated depletion at 24 h post-HIV-1 transduction but not at 48 h post-transduction [36]. The loss of UNG2 at the later time point was attributed to transcriptional repression by HIV-1 Vpr [120]. In light of the aforementioned structural findings, however, it is possible that HIV-1 Vpr binding to UNG2 over time favored the switch to an active dimer, thereby contributing to the endogenous UNG2 loss, independent of neddylation. If physiologically relevant, would this mechanism be applicable to all proteins targeted by Vpr/x or would it be limited to the subset that, like UNG2, are native substrates of CRL4-DCAF1? Of note, HIV-1 Vpr can also oligomerize [236]. How might Vpr multimers interplay with CRL4-DCAF1? 

In addition to neddylation and multimerization states, CRL4 may be post-translationally regulated in ways that have yet to be elucidated. For example, the C-terminus of both Cul4 types contain residues that would be predicted to be sites of phosphorylation [237]. How might these potential modifications influence Vpr/x function?

### 5.2. Taking Advantage of DCAF1′s Moonlighting

As discussed in Section 2.2, DCAF1 performs a subset of functions that appear to be independent of CRL4. DCAF1 has intrinsic kinase activity that can be blocked with B32B3, a small molecule inhibitor [58]. Do Vpr/x take advantage of this activity? Would B32B3 provide a tool to study this?

The discovery of DCAF1’s ability to also act as an adapter for the EDVP HECT E3 ubiquitin ligase raised the possibility that Vpr/Vpx could perform additional functions through this alternative complex. Indeed, at least two targets, TERT and CP110 (discussed in Section 3.6), have been identified as targets of HIV-1 Vpr via EDVP, and it is likely that additional targets will be revealed in time. Considering that both DCAF1 and DDB1 engage this alternative complex, future work aiming to characterize a novel Vpr/Vpx function should aim to distinguish between CRL4 and EDVP, as DCAF1 depletion alone may no longer suffice to claim function through CRL4. Of note, like what has been observed with several CRL4-DCAF1-mediated targets of HIV-1 Vpr, TERT and CP110 are native substrates of EDVP whose turnover is enhanced by HIV-1 Vpr, a theme expanded upon next.

### 5.3. The Benefits of Getting to Know Your Neighbors

First, it is worthwhile to note that a number of DCAF1-*independent* functions of HIV Vpr and Vpx have been identified and others are likely yet to be discovered. Some of these functions are briefly mentioned in Section 1.5, 3.4, and 4.3 and are reviewed in the context of CD4^+^ T-cell infection in [238]. While the majority of HIV Vpr and Vpx functions rely on DCAF1, the existence of these DCAF1-independent functions further underscores the versatility of these viral proteins.

Most of the functions ascribed to Vpr/x, especially HIV-1 Vpr, are directly linked to the normal physiological functions of CRL4-DCAF1. Namely cell cycle, DNA repair, chromatin remodeling, and gene expression. Indeed, the majority of the targets identified have been found to play a role in one or more of these normal cellular processes. Further, several of the targets discussed have been found to, at the very least, associate with DCAF1 (e.g., SLX4, ExoI, HLTF, and TASOR) in the absence of Vpr/x, and many have been confirmed as native substrates of CRL4-DCAF1 under normal physiological conditions (e.g., UNG2, TET2, and MCM10). Thus, Vpr and Vpx’s co-option of DCAF1 enables proximal access to a multitude of cellular proteins whilst simultaneously providing a convenient mechanism for disposal of any deemed detrimental to viral replication. Though not all targets identified appear to be native substrates of, or normally associate with, DCAF1 (e.g., SAMHD1), it is possible that “crosstalk” between intracellular pathways enables transient opportunities for Vpr/x to access targets that are not consistently within the vicinity of CRL4-DCAF1.

Greenwood et al. used a pulsed-Stable Isotope Labeling with Amino acids in Cell culture (pulsed SILAC) complementary proteomics analysis to reveal striking and widespread changes in the entire cellular protein content caused by HIV-1 Vpr. The SILAC method is described in [239] and demonstrated in [240]. Interestingly, no other HIV-1 viral protein, nor the HIV-2 paralogs Vpr or Vpx, were capable of eliciting such extensive changes. While several formerly validated HIV-1 Vpr targets were confirmed by this study (e.g., HLTF, MCM10, UNG2, TET2, and MUS81, etc.), this work also identified a number of other targets that had not previously been studied in the context of HIV-1 Vpr function. SMN1, CDCA2, and ZNF267, for example, were flagged as targets for HIV-1 Vpr and were found to contribute to the G2 cell cycle arrest phenotype. As described in the preceding sections, several proteins have been implicated in HIV-1 Vpr-mediated G2 cell cycle arrest. Thus, the discovery of these additional contributors implies that G2 cell cycle arrest is likely due to the degradation of multiple factors rather than a single target. Interestingly, induction of G2 cell cycle arrest accounted for only a modest proportion of the proteomic changes triggered by HIV-1 Vpr. Instead, the majority of the changes were mapped to the depletion of nuclear proteins with nucleic acid binding activity. This is consistent with the insert loop of HIV-1 Vpr mimicking DNA to recruit and degrade DNA-binding proteins (e.g., UNG2 and HLTF) (described in Section 3.1). Importantly, at least 13 proteins that co-immunoprecipitated with HIV-1 Vpr were reported to associate with DCAF1 in the absence of Vpr. Further, DCAF1 profoundly influenced the ability of HIV-1 Vpr to induce these widespread proteomic changes, dramatically emphasizing the advantage of usurping CRL4 [102]. Of note, the stringent criteria employed by this work, coupled with exclusive use of the CEM-T4 CD4^+^ T-cell line, suggests a larger number of proteins are targets of HIV-1 Vpr than have thus far been identified. HIV-1 Vpr is abundantly packaged into HIV-1 virions, and while recent studies place capsid uncoating at the nuclear pore or within the nucleus [138], both HIV-1 Vpr [241] and HIV-2 Vpx [242] have been shown to dissociate from the viral core prior to uncoating through an unestablished mechanism. Therefore, upon entry into the cell Vpr is poised to engage multiple targets to both safeguard early replication events as well as prime the cell for maximum virus production. 

### 5.4. A Macrophage Is Not a Macrophage Is Not a Macrophage

While most myeloid lineage cells share a common hematopoietic stem cell origin, considerable heterogeneity emerges as a result of tissue-specific signals. Thus, though macrophages and dendritic cells share a common role as APCs, their functional and biochemical overlap is limited. Indeed, a spectrum of characteristics and phenotypes are used to define a “macrophage” versus a “dendritic cell,” and each term broadly describes a range of cells that can be further subcategorized (reviewed in [243]). The same principle applies to CD4^+^ T-cells (extensively reviewed in [244]) and all cellular targets of HIV in the context of human infection (reviewed in [131]). These differences are likely reflected in the seemingly contradictory, incomplete, or difficult-to-interpret findings that plague Vpr research. It is assumed that studies utilizing primary immune cells are the most relevant, and several of the findings described here have yet to be validated in primary immune cells, particularly CD4^+^ T-cells (reviewed in [238]). Use of primary immune cells, however, does not guarantee uniformity of results among laboratories or necessarily mirror human infection. Similarly, results obtained in model cell lines or in vitro analyses do not exclude those findings from having relevance to human infection. Thus, two seemingly disparate findings may both be true under the right cellular context. For example, the apparent discrepancies in cellular targets that enable Vpr-mediated G2 cell cycle arrest may all be functionally valid in human infection. Though this conserved function of Vpr modestly impacts cycling CD4^+^ T-cell infection in cell culture, induction of G2 cell cycle arrest may be so critical to HIV replication in human infection that Vpr-evolved promiscuous use of CRL4-DCAF1 to ensure arrest occurs by whatever means available, regardless of the intracellular environment the virus finds itself in. This same concept may hold true with other Vpr/x phenotypes.
